# Novel fatty acid methyl esters from the actinomycete *Micromonospora aurantiaca*

**DOI:** 10.3762/bjoc.7.200

**Published:** 2011-12-20

**Authors:** Jeroen S Dickschat, Hilke Bruns, Ramona Riclea

**Affiliations:** 1 Institut für Organische Chemie, Technische Universität Braunschweig, Hagenring 30, 38106 Braunschweig, Germany

**Keywords:** actinomycetes, FAMEs, fatty acid biosynthesis, GC–MS, volatiles

## Abstract

The volatiles released by *Micromonospora aurantiaca* were collected by means of a closed-loop stripping apparatus (CLSA) and analysed by GC–MS. The headspace extracts contained more than 90 compounds from different classes. Fatty acid methyl esters (FAMEs) comprised the major compound class including saturated unbranched, monomethyl and dimethyl branched FAMEs in diverse structural variants: Unbranched, α-branched, γ-branched, (ω−1)-branched, (ω−2)-branched, α- and (ω−1)-branched, γ- and (ω−1)-branched, γ- and (ω−2)-branched, and γ- and (ω−3)-branched FAMEs. FAMEs of the last three types have not been described from natural sources before. The structures for all FAMEs have been suggested based on their mass spectra and on a retention index increment system and verified by the synthesis of key reference compounds. In addition, the structures of two FAMEs, methyl 4,8-dimethyldodecanoate and the ethyl-branched compound methyl 8-ethyl-4-methyldodecanoate were deduced from their mass spectra. Feeding experiments with isotopically labelled [^2^H_10_]leucine, [^2^H_10_]isoleucine, [^2^H_8_]valine, [^2^H_5_]sodium propionate, and [*methyl*-^2^H_3_]methionine demonstrated that the responsible fatty acid synthase (FAS) can use different branched and unbranched starter units and is able to incorporate methylmalonyl-CoA elongation units for internal methyl branches in various chain positions, while the methyl ester function is derived from *S*-adenosyl methionine (SAM).

## Introduction

Lipids in general, and particularly fatty acids (FAs), belong to the most important building blocks of biological systems. They fulfill various physiological functions, such as cell-membrane assembly or, as highly reduced carbon compounds, energy storage, and are therefore found in every single living cell on earth. In bacteria the cell membranes are mainly formed from phospholipids such as phosphatidylcholines that contain a FA diglyceride, a phosphate, and a phophate-bound choline. The simplest type of phospholipid is made up from unbranched saturated FAs with typical chain lengths of 16 or 18 carbon atoms, but sometimes also shorter or longer FAs can be found. The fluidity of bacterial cell membranes can be tuned, e.g., by the introduction of methyl branches or olefinic double bonds [1].

**Scheme 1:**
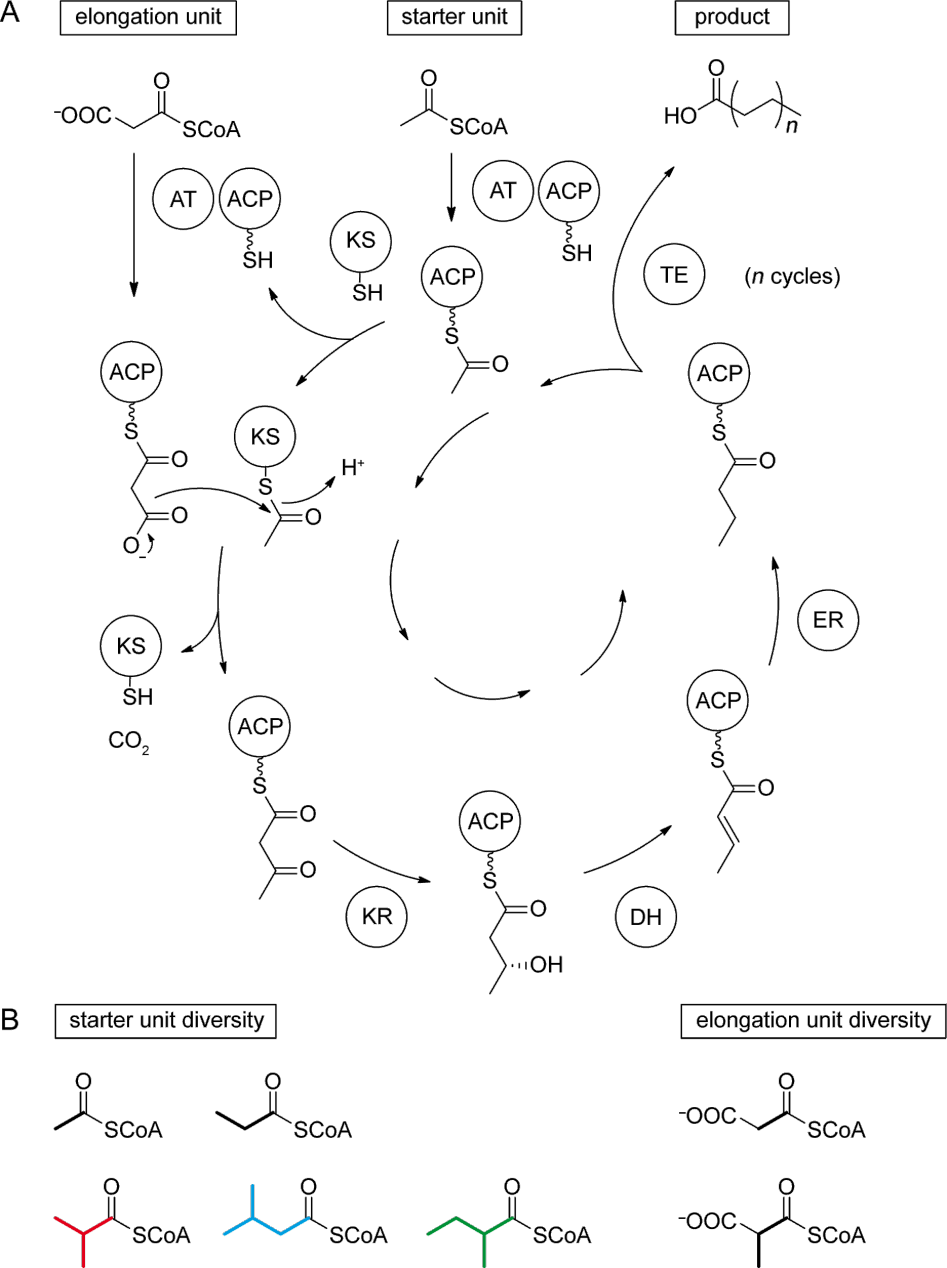
Fatty acid biosynthesis.

The biosynthesis of FAs is a repetitive chain elongation process catalysed in animals and fungi by multifunctional megasynthases, and in plants or bacteria by a set of discrete enzymes with equal functions to the individual and respective megasynthase domains. In both cases a starter unit, usually acetyl-CoA, is selected by the acetyl transferase (AT) and loaded onto an acyl-carrier-protein (ACP), or, more precisely, onto the thiol end of a phosphopantetheinyl linker that is attached to a highly conserved serine residue of the ACP (Scheme 1A). The acetyl moiety is then taken over by a conserved cystein residue of the ketosynthase (KS) making the ACP in turn available for the uptake of an elongation unit, in most cases malonyl-CoA, which is again selected and transferred by the AT. The reaction between the ACP-bound malonyl and the KS-bound acetyl group under decarboxylation conditions results in the formation of acetoacetyl-S~ACP by release of the KS. A three-step reductive process involving the subsequent actions of a ketoreductase (KR), a dehydratase (DH), and an enoyl reductase (ER) yields butyryl-S~ACP via (*R*)-3-hydroxybutyryl-S~ACP and crotyl-S~ACP. In summary of these transformations, the starter unit is elongated by two fully reduced carbon atoms, and *n* iterations of this elongation procedure yield a fatty acyl-S~ACP product with a chain length of (2*n* + 2) carbon atoms, with the final chain length being solely dependent on the size of the acting FAS’s active site (although FA biosynthesis is catalysed by several discrete enzymes in bacteria, the term FAS, strictly speaking short for fatty acid synthase and thereby implying the action of only one single enzyme, will be used here for the complete bacterial FA biosynthetic machinery for reasons of brevity and simplicity). Product release from the ACP is achieved by action of a thioesterase (TE) to provide the unbound FA. The combination of an acetyl-CoA starter and malonyl-CoA elongators always leads to unbranched FAs with an even number of carbon atoms (even FAs). Structural variations are possible through the use of alternative starters, such as propionyl-CoA, for the synthesis of odd FAs (Scheme 1B). The branched amino acids valine and leucine provide, by transamination and oxidative decarboxylation, the *iso*-branched starters isobutyryl-CoA (red) for *iso*-even FAs and isovaleryl-CoA (blue) for *iso*-odd FAs, whereas the same reactions from isoleucine yield (*S*)-2-methylbutyryl-CoA (green) for *anteiso*-odd FA biosynthesis. Internal methyl branches can be introduced through the use of methylmalonyl-CoA elongation units, and occur due to the logic of FA biosynthesis in even-numbered positions of the FA carbon chain. An alternative mechanism leading to the same methyl branching pattern is well-known from polyketide biosynthesis and involves the incorporation of a malonyl-CoA unit followed by SAM-dependent methylation of the new α-carbon. Further alternative starters are known [2–4], but these cases are rare. In contrast, the usage of alternative elongation units such as ethylmalonyl-CoA [5], propylmalonyl-CoA [6], isobutylmalonyl-CoA [7], or methoxymalonyl-ACP [8] remains almost limited to polyketide synthesis and is only found in very exceptional cases of fatty acid biosynthesis [9].

**Figure 1:**
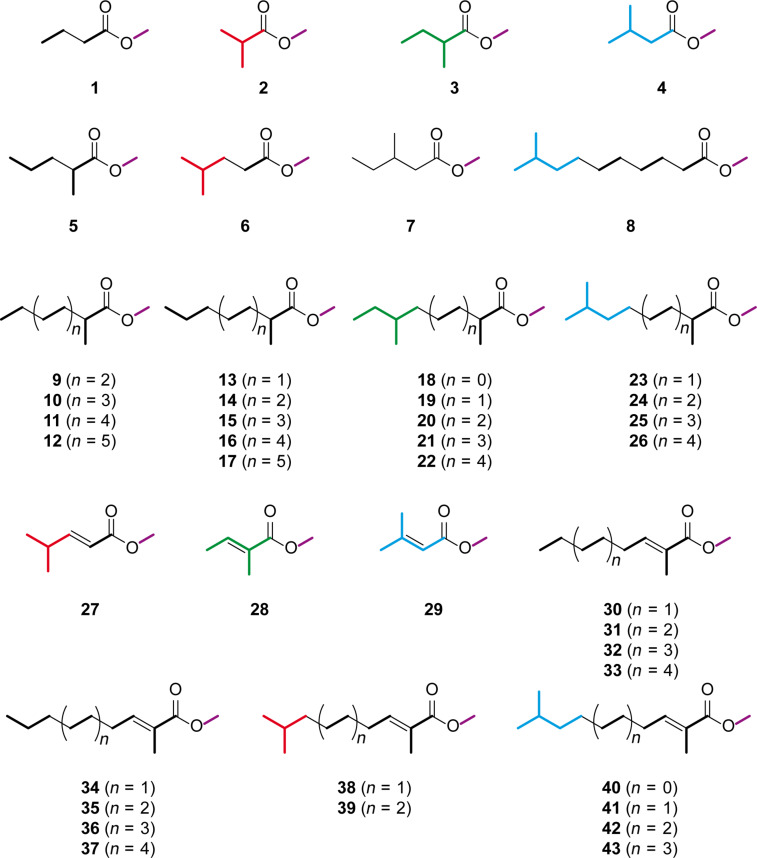
Volatile methyl esters from bacteria.

Due to their ability to participate in hydrogen bonds and to form stabilised dimers, carboxylic acids have relatively low vapour pressures and, therefore, high boiling points. The volatility of carboxylic acids can be increased by their transformation into methyl esters, e.g., compare the boiling points of acetic acid (bp 118 °C) and methyl acetate (bp 57 °C). Some bacteria can methylate FAs to yield the corresponding methyl esters, resulting not only in an increased volatility, but making them at the same time unavailable for other biosynthetic transformations. Such fatty acid methyl esters (FAMEs) have previously been reported as headspace constituents of diverse bacteria [10]. The saturated compounds methyl butanoate (**1**), methyl isobutyrate (**2**), methyl 2-methylbutyrate (**3**), methyl isovalerate (**4**), methyl 2-methylpentanoate (**5**), methyl isocaproate (**6**), and methyl 3-methylpentanoate (**7**) were found in actinomycetes [11–12]. Methyl 9-methyldecanoate (**8**) is released by the myxobacterium *Stigmatella aurantiaca* DW4/3-1 [13]. A complex mixture of several methyl 2-methylalkanoates (**9**–**26**) was recently reported from the gliding bacterium *Chitinophaga* Fx7914 [14]. Some α,β-unsaturated FAMEs have also been described, such as methyl 4-methylpent-2-enoate (**27**), methyl tiglate (**28**), and methyl 3-methylcrotonate (**29**) from actinomycetes [12], and various methyl 2-methylalk-2-enoates (**30**–**43**) from *Chitinophaga* [14]. The proposed building blocks for the biosynthesis of these methyl esters are highlighted in bold and by use of a colour code in Figure 1. For the methyl 2-methylalkanoates and -alk-2-enoates from *Chitinophaga*, the origin of the methyl group from *S*-adenosyl methionine (SAM, purple) and of the 2-methyl groups from methylmalonyl-CoA was determined by feeding experiments [14].

During our ongoing analysis of the volatiles released by different bacteria and fungi with high potential for secondary metabolism, the actinomycete *Micromonospora aurantiaca* ATCC 27029 came to our attention. This gram-positive, sporulating bacterial genus includes producers of important antibiotics such as the aminoglycoside gentamycin [15] and the antitumor antibiotics lomaiviticins A and B (*Micromonospora lomaivitiensis*) [16]. Here the results of the headspace analyses of *M. aurantiaca* are described. Besides compounds from other classes, several methyl esters were identified. The identification of these esters from their mass spectra and retention indices, as well as the verification of the proposed structures by synthesis of representative reference compounds is presented.

Besides several other compounds, such as terpenes, pyrazines, aromatic compounds and methyl ketones, more than half of the components identified are unbranched and mono- and dimethylbranched FAMEs, many of this last group having not been reported before.

## Results and Discussion

### Volatiles from *Micromonospora aurantiaca*

**Figure 2:**
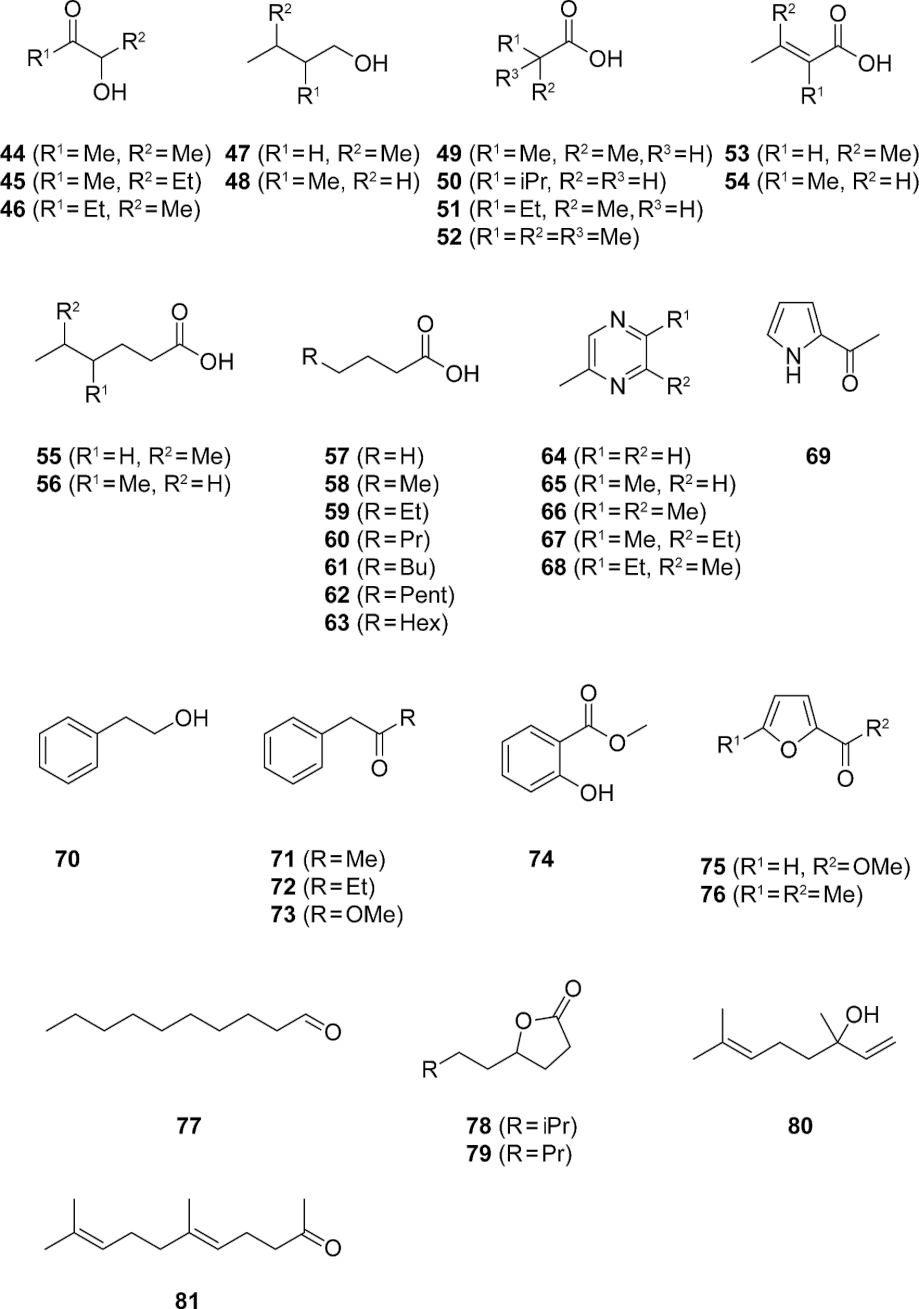
Compounds found in the headspace extracts of *M. aurantiaca*.

**Figure 3:**
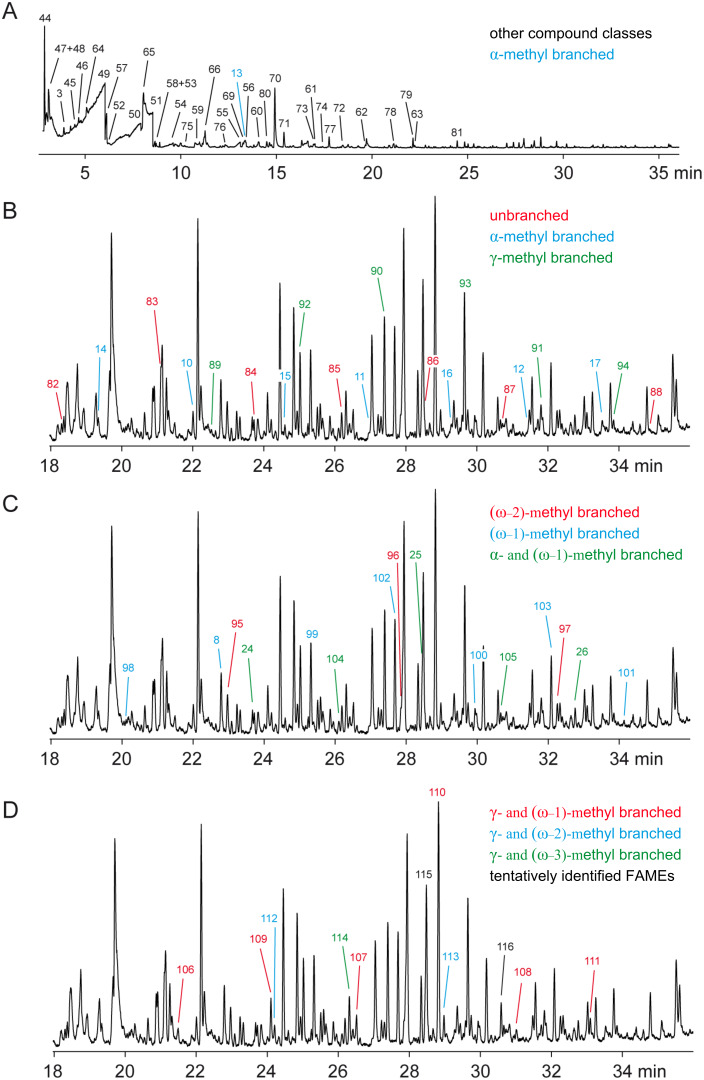
Total ion chromatograms of the headspace extract from *M. aurantiaca* (A), and expansions of the total ion chromatogram (18–36 min) showing the relevant peaks of the FAMEs (B–D, for clarity the same expansion is shown in three replicates for FAMEs from different series). The colour code used for these series of FAMEs is given in the respective figures.

The volatiles released by agar plate cultures of the actinomycete *M. aurantiaca* ATCC 27029 were collected by use of a closed-loop stripping apparatus (CLSA), as described previously [17–18], and the obtained headspace extracts were analysed by GC–MS. The structures of the identified compounds (apart from FAMEs, which will be discussed below) are shown in Figure 2, the chromatogram of a representative sample is presented in Figure 3A, and the full results from two extracts are summarised in Table S1 of Supporting Information File 1. *M. aurantiaca* released more than 90 compounds from different compound classes including carboxylic acids, FAMEs, lactones, alcohols, aldehydes, acyloins, nitrogen and oxygen heterocycles, aromatic compounds, and terpenoids. Carboxylic acids were dominating, and this class was composed of the branched isobutyric acid (**49**), isovaleric acid (**50**), and 2-methylbutyric acid (**51**) as the main compounds, with minor amounts of 5-methylhexanoic acid (**55**) and 4-methylhexanoic acid (**56**), the α,β-unsaturated 3-methylbut-2-enoic acid (**53**) and 2-methylbut-2-enoic acid (**54**), a homologous series of unbranched saturated compounds from butyric acid to decanoic acid (**57**–**63**), and the unusual 2,2-dimethylpropanoic acid (**52**). Compound **51** was accompanied by minor amounts of its methyl ester **3**. The acids **49**–**51** can arise from the amino acids valine, leucine, and isoleucine, respectively, by transamination and oxidative decarboxylation, whereas **52** is equally available from the unusual *tert*-leucine. The acids **53** and **54** can arise from their saturated counterparts **50** and **51** by dehydrogenation. A two-carbon elongation of **50** and **51** by means of the fatty acid biosynthetic pathway can generate **55** and **56**, while their reduction to the respective alcohols provides **47** and **48**. Acetoin (**44**) was also found as a major compound, together with its derivatives 3-hydroxypentan-2-one (**45**) and 2-hydroxypentan-3-one (**46**). Acyloins were recently identified as precursors for pyrazines from *Corynebacterium glutamicum* in our laboratory [19]. The *M. aurantiaca* headspace extracts contained methylpyrazine (**64**), 2,5-dimethylpyrazine (**65**), trimethylpyrazine (**66**), 2-ethyl-3,6-dimethylpyrazine (**67**), and 2-ethyl-3,5-dimethylpyrazine (**68**), which may also arise from acyloins. Further identified compounds were 2-acetylpyrrole (**69**), 2-phenylethanol (**70**), phenylacetone (**71**), 1-phenylbutan-2-one (**72**), methyl phenylacetate (**73**), methyl salicylate (**74**), methyl furan-2-carboxylate (**75**), 2-acetyl-5-methylfuran (**76**), decanal (**77**), 7-methyloctan-4-olide (**78**), nonan-4-olide (**79**), and the terpenoids linalool (**80**) and geranyl acetone (**81**).

**Figure 4:**
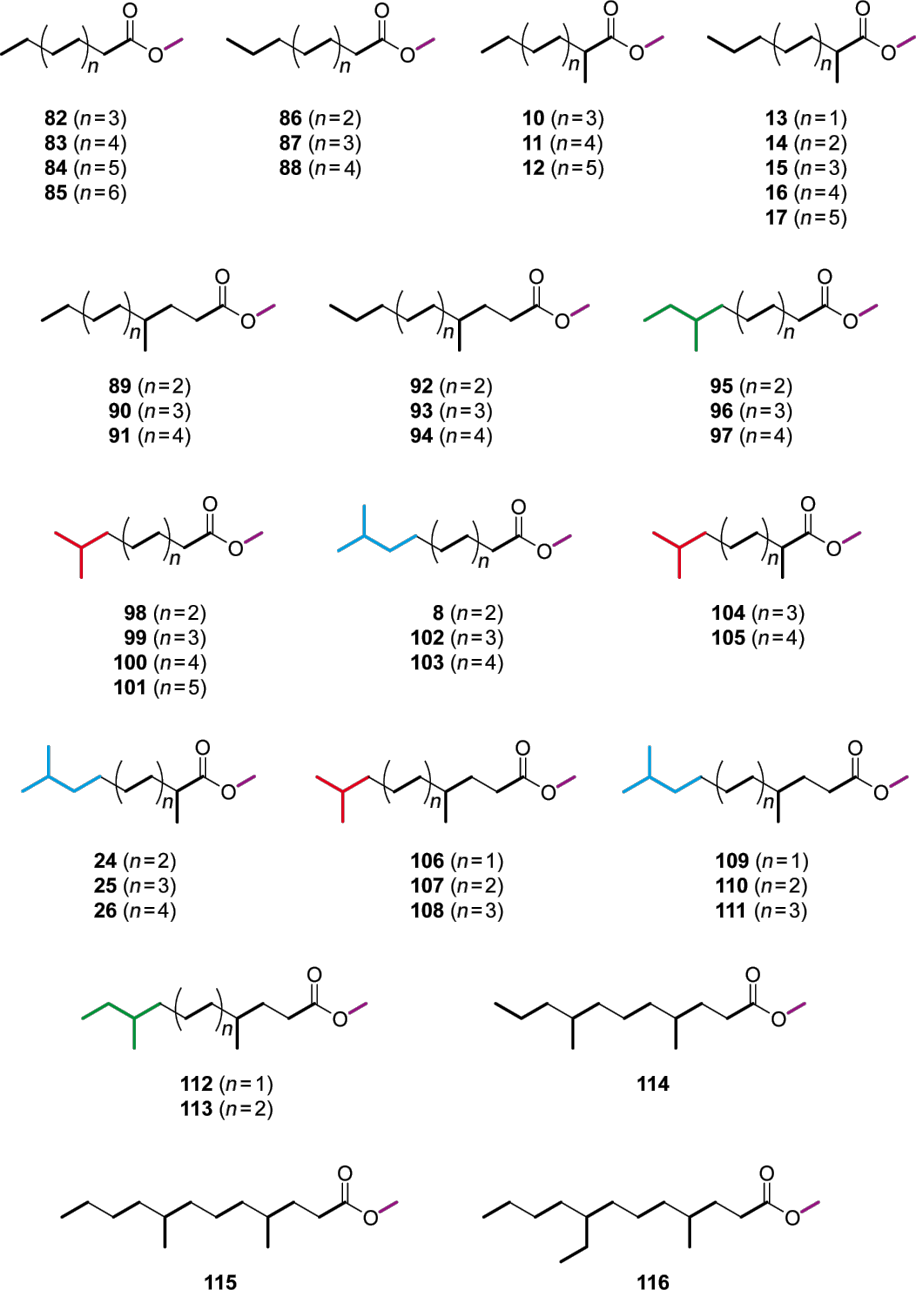
FAMEs identified in the headspace extracts from *M. aurantiaca*.

**Figure 5:**
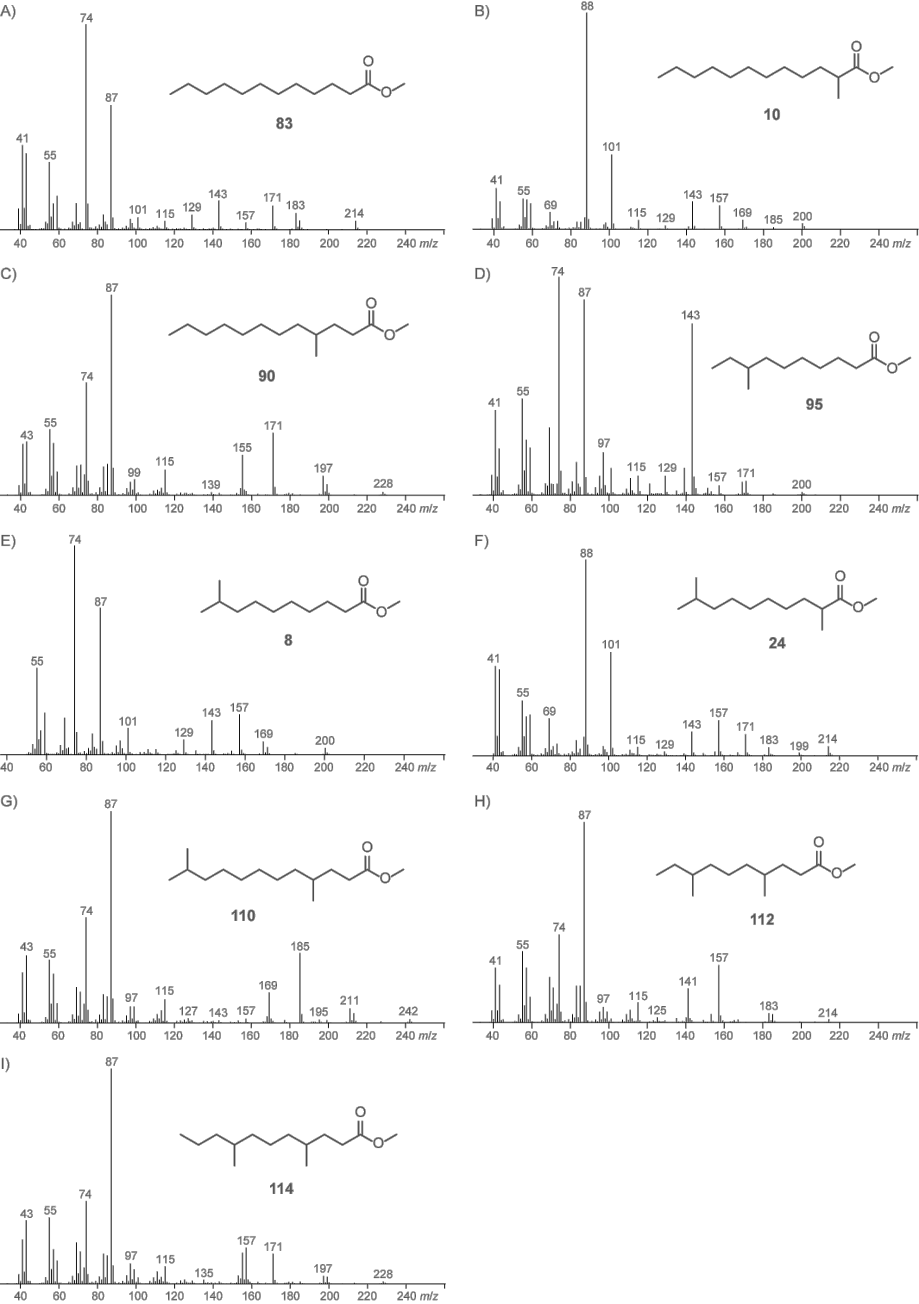
Mass spectra of (A) methyl dodecanoate (**83**), (B) methyl 2-methyldodecanoate (**10**), (C) methyl 4-methyldodecanoate (**90**), (D) methyl 8-methyldecanoate (**95**), (E) methyl 9-methyldecanoate (**8**), (F) methyl 2,9-dimethyldecanoate (**24**), (G) methyl 4,11-dimethyldodecanoate (**110**), (H) methyl 4,8-dimethyldecanoate (**112**), and (I) methyl 4,8-dimethylundecanoate (**114**).

**Scheme 2:**
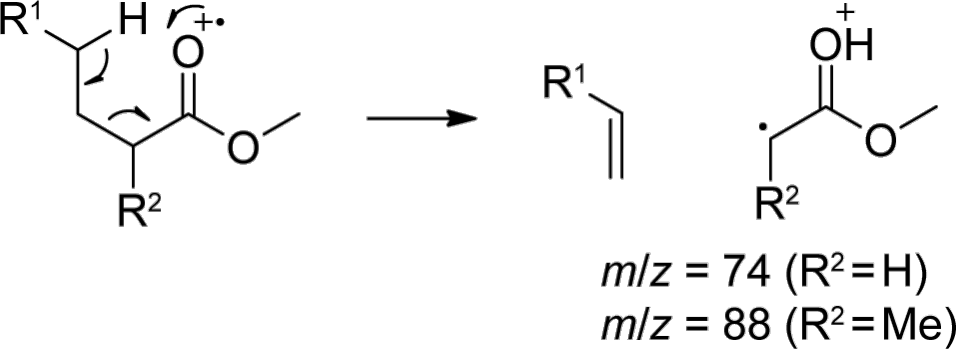
McLafferty fragmentation of FAMEs.

Besides the compounds mentioned above, several saturated FAMEs were present in the headspace extracts (Figure 4). All the identified FAMEs were divided into groups according to their pattern of methyl branchings. Unbranched FAMEs (Figure 3B, red) included all even and odd members of the homologous series from methyl nonanoate to methyl tetradecanoate, in addition to methyl hexadecanoate. These compounds were readily identified from their mass spectra by comparison to library spectra and subsequent GC–MS analysis of synthetic standards. Mass spectra of unbranched FAMEs (for mass spectrum of methyl dodecanoate see Figure 5A) are characterised by fragment ions at *m*/*z* = 74 (McLafferty rearrangement, Scheme 2), *m*/*z* = 87 (β-cleavage), and [M − 31]^+^ (loss of OMe). Further fragment ions [M − C*_n_*H_2_*_n_*_+1_]^+^ arise from cleavage of the saturated unbranched alkyl chain.

All other FAMEs were isomers of these unbranched compounds and were assumed to be methyl branched FAMEs due to biosynthetic considerations as outlined above. The structures of these branched compounds have been suggested based on careful analysis of their mass spectra and on a modified retention-index increment system [20]. Following this system, the retention index *I*_calc._ of a methyl branched compound can be calculated (Equation 1) by

[1]
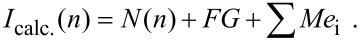


**Figure 6:**
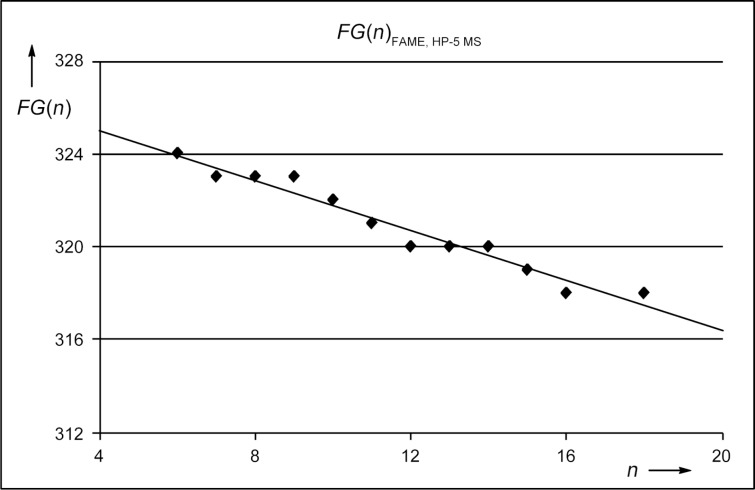
The functional group increment *FG*(n)_FAME, HP-5 MS_.

The increment *N*(*n*) depends on the number of carbon atoms *n* in the longest alkyl chain and is *N*(*n*) = 100 *n*, *FG* is an increment for the functional group, and the increments *Me*_i_ have to be considered for methyl branches depending on the positions i of branching. The increments *FG* and *Me*_i_ have to be determined for each type of GC column. In a first approximation, these increments can be assumed to be constants, but as will be discussed below both *FG*(*n*) and *Me*_i_(*n*) are slightly dependent on the length of the alkyl chain, giving better results for the calculated retention indices if this dependency is considered. For all of the following analyses the functional group increment for FAMEs on a HP-5 MS column (*FG*(*n*)_FAME, HP-5 MS_) was determined from the homologous series of unbranched FAMEs (Table S2 and Figure S1 of Supporting Information File 1). By linear regression (Figure 6) the functional group increment (Equation 2) was

[2]
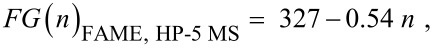


resulting in a modified Equation 1

[3]
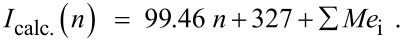


**Scheme 3:**
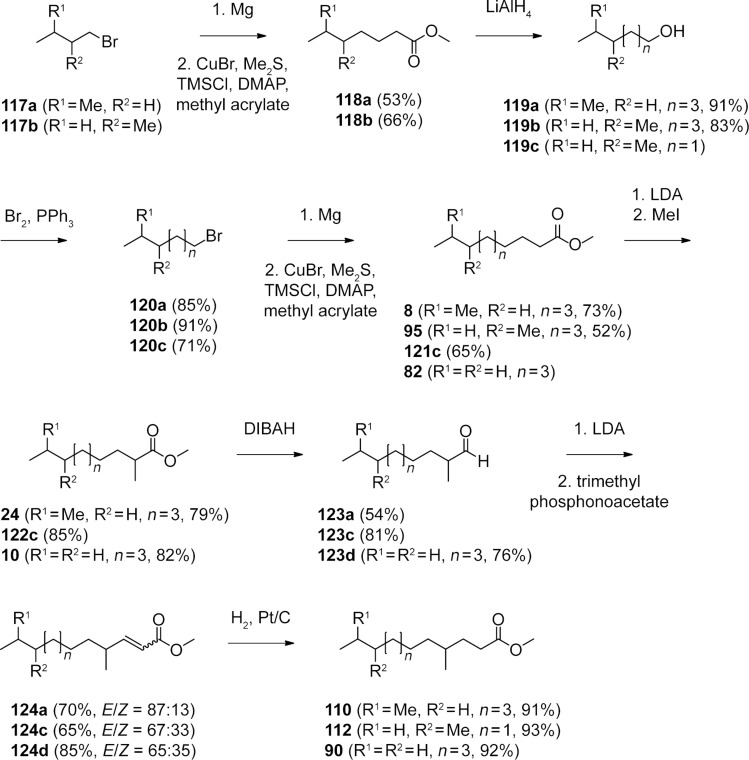
Synthesis of FAMEs identified from *M. aurantiaca*.

A series of compounds (Figure 3A and Figure 3B, blue) exhibited mass spectra with two significant fragment ions at *m*/*z* = 88 as the base peak and at *m*/*z* = 101, indicating a methyl branching in a α-position (Scheme 2, cf. Figure 5B for mass spectrum of methyl 2-methyldecanoate). For the determination of the increment *Me*_α_ the reference compound methyl 2-methyldecanoate (**10**) was synthesised by α-alkylation of **82** (Scheme 3). The mass spectrum and retention index (*I* = 1357) of the product were identical to those of the natural compound. By using Equation 3 the increment for a methyl branching in a α-position was determined as *Me*_α_ = 35, resulting in the calculated retention indices for the α-methyl branched FAMEs as listed in Table S3 of Supporting Information File 1, column 4. The calculated retention indices fitted perfectly for compounds with a chain length of around 10 carbon atoms, which is not surprising since *Me*_α_ was determined from **10**, but the indices deviated slightly from the measured values for shorter (*n* = 7: *I*_nat._ − *I*_calc._ = 4) or longer (*n* = 15: *I*_nat._ − *I*_calc._ = −2) FAMEs. In other words, *Me*_α_ was dependent on the chain length. A linear regression analysis gave

[4]
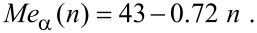


Recalculation of the retention indices of the α-methyl branched FAMEs, taking into account the dependency of *Me*_α_ on the chain length, resulted in the values listed in Table S3 of Supporting Information File 1, column 5, which perfectly fitted the measured retention indices.

The next class of compounds (Figure 3B, green) showed characteristic fragment ions at *m*/*z* = 87 and *m*/*z* = 74 similar to the unbranched FAMEs, but in contrast the β-cleavage was more important than the McLafferty fragmentation (cf. Figure 5C for mass spectrum of methyl 4-methyldodecanoate). This, together with an almost completely missing fragment ion at *m*/*z* = 101, accounting for a γ-cleavage in unbranched FAMEs, suggested the presence of a methyl group in a γ-position, which leads to a γ-fragmentation with *m*/*z* = 115. A synthesis of methyl 4-methyldodecanoate (**90**) was performed starting from **10** (Scheme 3). Reduction to the aldehyde **123d** and subsequent Horner–Wadsworth–Emmons reaction gave the α,β-unsaturated ester **124d**, which upon catalytic hydrogenation yielded **90**. The synthetic compound was identical to the natural FAME as judged by mass spectrum and retention index (*I* = 1572). The retention index of this reference compound was used for to determine that *Me*_γ_ = 51, resulting in the calculated retention indices for all γ-methyl branched FAMEs as summarised in Table S4 of Supporting Information File 1. Correction of the increment *Me*_γ_ by linear regression gave

[5]
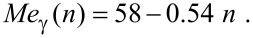


The corrected calculated retention indices, taking Equation 5 into consideration, were in good agreement with the measured retention indices.

Another group of FAMEs (Figure 3C, red) showed mass spectra with significant fragment ions at *m*/*z* = 74 and *m*/*z* = 87, like the unbranched compounds (for mass spectrum of methyl 8-methyldecanoate cf. Figure 5D), and were assumed to be branched towards the Me terminus, i.e., these FAMEs are suggested to originate from a branched amino acid starter. Within this group only the FAMEs derived from the odd FAs were found, pointing to an isoleucine-derived starter and a methyl branching in the (ω−2)-position. Furthermore, this suggestion was supported by the intensity of the [M − 57]^+^ ion, which is typical for *anteiso*-FAMEs. The synthesis of methyl 8-methyldecanoate (**95**) as a reference compound was started from 1-bromo-2-methylbutane (**117b**, Scheme 3). Copper-catalysed 1,4-addition of the respective Grignard reagent to methyl acrylate in the presence of trimethylchlorosilane, dimethyl sulfide, and 4-dimethylaminopyridine gave methyl 5-methylheptanoate (**118b**). A sequence of LiAlH_4_ reduction to the alcohol **119b**, conversion into the bromide **120b**, and Cu-mediated 1,4-addition of the Grignard reagent to methyl acrylate furnished the desired FAME **95**. Its mass spectrum and retention index (*I* = 1392) were in good agreement with those of the natural product. The increment *Me*_ω−2_ = 70 was determined from the retention index of compound **95**. In contrast to the increments *Me*_α_ and *Me*_γ_ the increment *Me*_ω−2_ proved to be largely independent of the chain lengths of the FAMEs and gave good results for the calculated retention indices over a wide range of *n*, including all other natural FAMEs of this type from *M. aurantiaca* and the compounds **118b** and **121c**, which were obtained in the syntheses of **95** and methyl 4,8-dimethyldecanoate (**112**) as described below (Table S5 of Supporting Information File 1). Furthermore, the value of *Me*_ω−2_ was in good agreement with a previously published value determined on a BPX-5 column (*Me*_ω−2_ = 73) [20].

The next series of FAMEs (Figure 3C, blue) also had very similar mass spectra compared to the unbranched and (ω−2)-methyl branched compounds dominated by the fragments at *m*/*z* = 74 and *m*/*z* = 87 (the mass spectrum of methyl 9-methyldecanoate is shown in Figure 5E). Within this series both even and odd FAMEs were detected, and therefore, these compounds were suggested to be (ω−1)-methyl branched and derived from leucine or valine starters, respectively. As typical for (ω−1)-methyl branched compounds, these FAMEs eluted slightly earlier than their (ω−2)-methyl branched counterparts. Additional support for this suggestion was given by the occurrence of relatively intense fragments at [M − 43]^+^, as can be expected for *iso*-FAMEs. In direct analogy to the synthesis of **95**, methyl 9-methyldecanoate (**8**) was synthesised from 1-bromo-3-methylbutane (**117a**, Scheme 3) and was identical to the volatile from *M. aurantiaca* in terms of mass spectrum and retention index (*I* = 1385), according to an increment of *Me*_ω−1_ = 63. The retention indices of all other volatiles from *M. aurantiaca* and of the intermediate **118a** in the synthesis of **8** were calculated from this constant increment and were in good agreement with the measured values (Table S6 of Supporting Information File 1).

In addition to these mono-methyl branched FAMEs, several classes of multiply methyl branched compounds were identified. The first group of compounds (Figure 3C, green) exhibited mass spectra that were very similar to those of the α-branched FAMEs described above, with dominating fragment ions at *m*/*z* = 88 and *m*/*z* = 101 (the mass spectrum of methyl 2,9-dimethyldecanoate is depicted in Figure 5F), but compounds of this class eluted significantly earlier than their mono-branched isomers, pointing to a second methyl branch within the alkyl chain, likely towards the methyl terminus. Since both even and odd compounds of this series were present, the structures of α- and (ω−1)-methyl branched FAMEs derived from leucine and valine starters, respectively, were suggested (Table S7 of Supporting Information File 1). The increments *FG*(*n*)_FAME, HP-5 MS_, *Me*_α_(*n*), and *Me*_ω−1_ as determined above were used for the calculation of retention indices. The calculated values were in perfect agreement with the measured values, thus corroborating the suggested structures. For unambiguous proof, an exemplary reference compound of this series was synthesised (Scheme 3). α-Methylation of **8** yielded methyl 2,9-dimethyldecanoate (**24**), which was identical to the respective FAME from *M. aurantiaca*.

A second series of dimethyl branched FAMEs (Figure 3D, red) exhibited very similar mass spectra to the γ-methyl branched esters (for mass spectrum of methyl 4,11-dimethyldodecanoate cf. Figure 5G). Within this class again both even and odd FAMEs were found, suggesting that they were derived from valine or leucine starters and therefore γ- and (ω−1)-methyl branched. Calculations of the retention indices for the suggested structures were in good agreement with the measured data (Table S8 of Supporting Information File 1), thus providing further evidence for the suggested structures. Final verification was obtained by synthesis of methyl 4,11-dimethyldodecanoate (**110**) from **24** by its reduction to the aldehyde **123a**, Horner–Wadsworth–Emmons olefination to **124a**, and catalytic hydrogenation (Scheme 3). The synthetic material was identical to the natural compound **110**.

A related group of compounds (Figure 3D, blue) proved to have very similar mass spectra, thus indicating a methyl group in a γ-position (Figure 5H shows the mass spectrum of methyl 4,8-dimethyldecanoate). The relatively high intensity of the [M − 57]^+^ fragment ion suggested the structures of *anteiso*-FAMEs, and accordingly, only the isoleucine-derived methyl esters of even-numbered FAs were found. Retention-index calculations (Table S9 of Supporting Information File 1) showed full agreement of the calculated retention indices with the experimental data. A synthesis of methyl 4,8-dimethyldecanoate (**112**) as a reference compound was carried out starting from 3-methylpentan-1-ol (**119c**), by similar methods as described above (Scheme 3). The alcohol **119c** was transformed into the bromide **120c**. The copper-catalysed Michael addition of the respective Grignard reagent to methyl acrylate yielded methyl 6-methyloctanoate (**121c**), which was α-methylated to **122c**. Reduction with DIBAH to the aldehyde **123c**, Horner–Wadsworth–Emmons olefination to **124c**, and final catalytic hydrogenation afforded **112**. The product exhibited the same mass spectrum and retention index (*I* = 1441) as the natural FAME. The slight deviations between the calculated and measured retention indices for compounds **106** (Δ*I* = 2) and **112** (Δ*I* = 3) may be attributed to the fairly close proximity of the two methyl branches, which are only three methylene units apart.

**Scheme 4:**
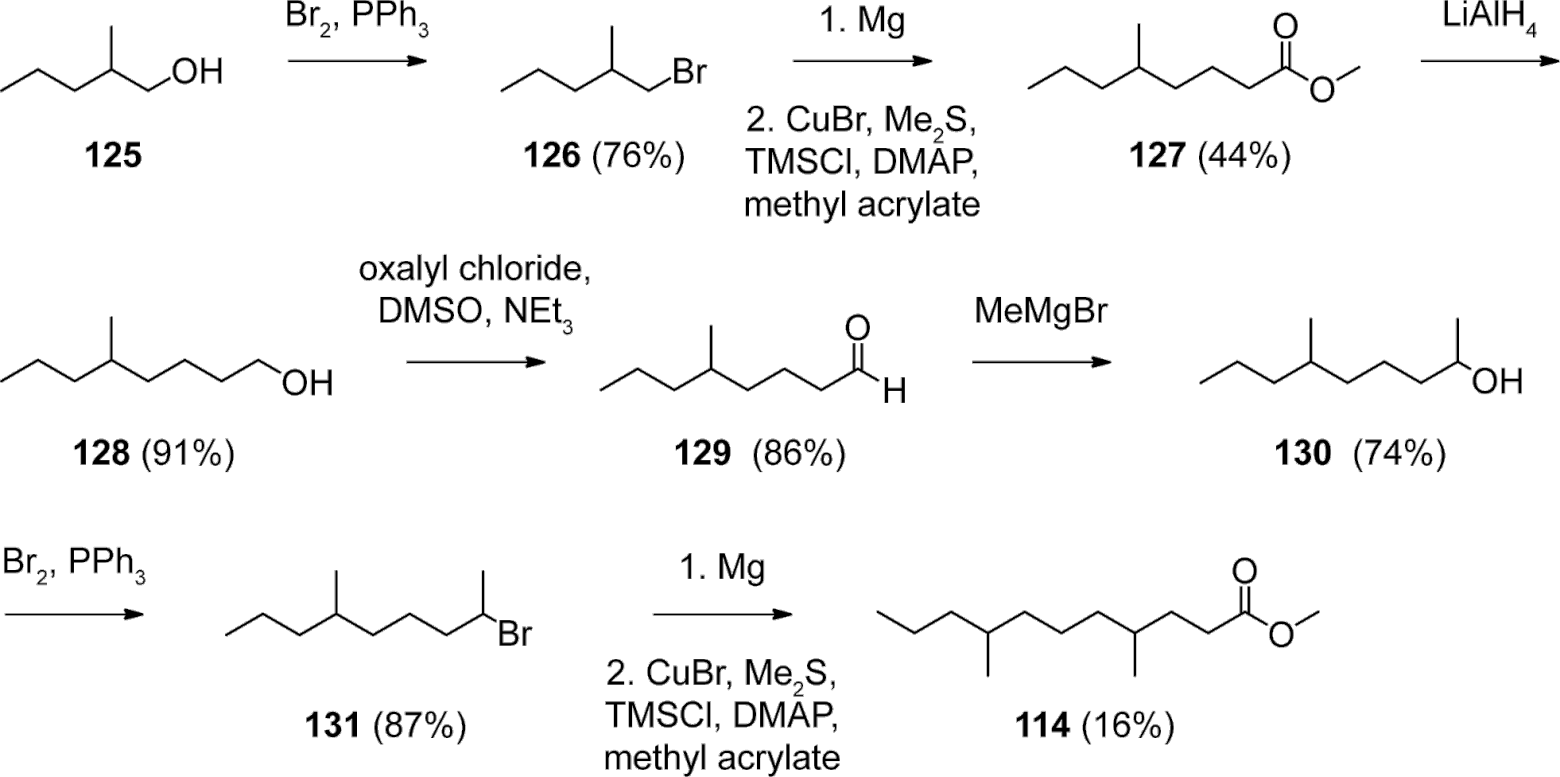
Synthesis of the γ- and (ω−3)-methyl branched FAME **114**.

The FAME **114** (Figure 3D, green) was also suggested to be γ-methyl branched based on its mass spectrum (the mass spectrum of methyl 4,8-dimethylundecanoate is presented in Figure 5I). This compound was an isomer of methyl 4-methyldodecanoate (**90**) and methyl 4,10-dimethylundecanoate (**107**), but eluted slightly earlier than **107**, resulting in a suggested structure of methyl 4,8-dimethylundecanoate. A synthesis of this compound was performed starting with bromination of 2-methylpentan-1-ol (**125**) and subsequent Cu-catalysed 1,4-addition of the respective Grignard reagent to methyl acrylate, resulting in methyl 5-methyloctanoate (**127**, Scheme 4). Reduction to the aldehyde **129** via the alcohol **128**, addition of methylmagnesium bromide, and bromination resulted in the secondary bromide **131**. Michael addition of its Grignard reagent to methyl acrylate was less efficient than for primary bromides, but gave the desired product **114** in low yield. The identity to the natural product was confirmed by comparison of GC–MS data.

**Figure 7:**
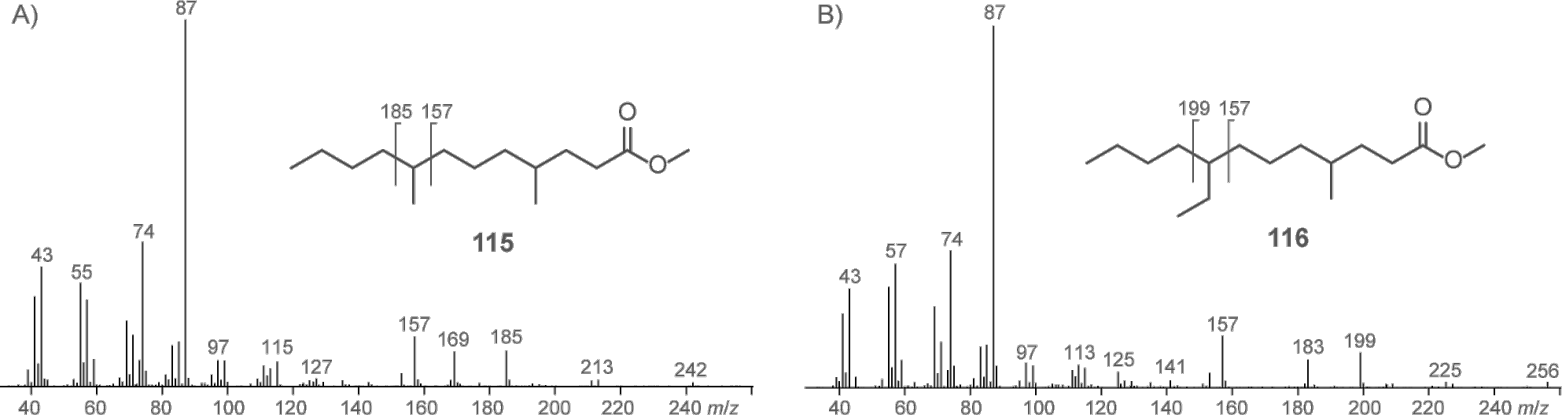
Mass spectra of tentatively identified methyl 4,8-dimethyldodecanoate (**115**) and methyl 8-ethyl-4-methyldodecanoate (**116**).

The structures of two additional FAMEs were suggested based on their mass spectra, but these were not verified by synthesis and therefore only tentatively identified (Figure 7). Both compounds were suggested to be γ-methyl branched due to the relative intensities of the fragment ions at *m*/*z* = 87 and *m*/*z* = 74. The first compound (Figure 7A) showed a molecular ion at *m*/*z* = 242 and further fragment ions at *m*/*z* = 157 and *m*/*z* = 185 resulting from the loss of C_4_H_9_ or C_6_H_13_, whereas no fragment ion accounting for the loss of C_5_H_11_ (*m*/*z* = 171) was observed. This pattern is in accordance with the structure of methyl 4,8-dimethyldodecanoate (**115**). The second compound (Figure 7B) exhibited a molecular ion at *m*/*z* = 256 and fragment ions at *m*/*z* = 157 and *m*/*z* = 199 according to the loss of C_4_H_9_ and C_7_H_15_, but not at *m*/*z* = 171 and *m*/*z* = 185 (loss of C_5_H_11_ and C_6_H_13_, respectively). This pattern suggested an ethyl branch in the 8-position corresponding to the structure of methyl 8-ethyl-4-methyldodecanoate (**116**). This compound is interesting in terms of its biosynthesis, because it may be formed by incorporation of an ethylmalonyl-CoA elongation unit. However, since no further compounds with ethyl branches were found, another biosynthetic option seems more likely. The compound 2-ethylhexanol is a widespread pollutant originating from plasticisers, and this compound could have been oxidised to 2-ethylhexanoic acid, transformed into its CoA derivative, and used as an unusual starter unit by the FAS to make **116**.

Several of the FAMEs emitted by *M. aurantiaca* are chiral, including, e.g., the α-, γ-, and (ω−2)-methyl branched FAMEs. In addition, compounds such as the γ- and (ω−2)-methyl branched FAMEs exist in two different diastereomers. However, these diastereomers, which were both contained in the synthetic material, e.g., of compounds **112** and **114**, were not separated on the HP5-MS column used for GC–MS analyses, and therefore the elucidation of the relative configurations was not possible, at least not by our GC approach. The only very small differences in the physical properties of these compounds were also reflected by the occurrence of only one set of signals in the NMR spectra of these mixtures of diastereomers. The separation of enantiomers of the chiral compounds described here on chiral GC columns is also a very hard task, especially for internally methyl branched FAMEs, and was beyond the scope of our work.

### Feeding experiments

To investigate the biosynthetic origin of the FAMEs, several feeding experiments with deuterated precursors were performed. These were directly added to the agar plate cultures and the headspace extracts were prepared by CLSA after ca. 2–3 days of growth. The CLSA extracts were then analysed by GC–MS. Incorporation of deuterated precursors was observable through the increased molecular masses and *m*/*z* ratios of certain fragment ions that could be used to localise the deuterium incorporation. One advantage of using deuterated precursors is that deuterium incorporation results in a decrease in the retention time of the analyte with respect to its unlabelled counterpart, i.e., the deuterated isotopomers are separated and their mass spectra can easily be interpreted [21].

#### Feeding of [^2^H_10_]isoleucine

One possible pathway to (ω−2)-methyl branched FAMEs is through the use of 2-methylbutyryl-CoA as a starting unit, which is available from isoleucine by transamination and oxidative decarboxylation. The alternative would be to use an acetyl-CoA starter followed by incorporation of a methylmalonyl-CoA elongation unit. The question as to which of these two alternatives is operative in *M. aurantiaca* was addressed by feeding of [^2^H_10_]isoleucine. In this feeding experiment *M. aurantiaca* produced large amounts of [^2^H_9_]-2-methylbutyric acid ([^2^H_9_]-**51**) and its respective methyl ester [^2^H_9_]methyl 2-methylbutyrate ([^2^H_9_]-**3**), both with incorporation rates >70%. The transamination of [^2^H_10_]isoleucine to 2-oxo-3-methylpentanoic acid proceeds with the loss of one deuterium, and accordingly, nine deuterium atoms were incorporated into **3**, as indicative by a shift of the molecular ion of **3** from *m*/*z* = 116 to *m*/*z* = 125 (compare Figures S2A and S2B of Supporting Information File 1). The fragment ion at *m*/*z* = 101, arising through the loss of a methyl group, shifted to *m*/*z* = 107, whereas no signal was detected at *m*/*z* = 110 in the mass spectrum of [^2^H_9_]-**3**. Therefore, the respective fragment ion only arises by methyl cleavage from the acyl moiety, and not by loss of the methyl group from the ester function. Further diagnostic fragment ions formed by α-cleavage (*m*/*z* = 57) and McLafferty rearrangement (*m*/*z* = 88) shifted to *m*/*z* = 66 and *m*/*z* = 93, in full agreement with the structure of [^2^H_9_]-**3**. For the respective free acid **51** no molecular ion is visible, but the fragment ion at *m*/*z* = 101, formed by loss of one hydrogen from the carboxylic acid function, was detected at *m*/*z* = 110 for [^2^H_9_]-**51**, indicating the incorporation of nine deuteriums (Figures S2C and S2D of Supporting Information File 1). Further fragment ions were observed at *m*/*z* = 66 (α-cleavage, + 9 amu), *m*/*z* = 79 (McLafferty rearrangement, + 5 amu), and *m*/*z* = 101 (loss of methyl group, + 6 amu), clearly establishing the identity of [^2^H_9_]-**51**. The uptake of isoleucine in the (ω−2)-methyl branched FAMEs was also observed with high incorporation rates (>90%) for methyl 12-methyltetradecanoate (**97**, Figures S2E and S2F of Supporting Information File 1), methyl 10-methyldodecanoate (**96**), and methyl 14-methylhexadecanoate (not shown). The mass spectrum of [^2^H_9_]-**97** is characterised by a molecular ion at *m*/*z* = 265 showing the incorporation of nine deuteriums, whereas the fragment ions at *m*/*z* = 74 and *m*/*z* = 87 indicative of the structure of a methyl ester are not shifted relative to the unlabelled material. The compound methyl 14-methylhexadecanoate was not found under the original experimental conditions without feeding of [^2^H_9_]isoleucine, demonstrating that the production of (ω−2)-methyl branched FAMEs by *M. aurantiaca* can be activated by isoleucine supply. Unfortunately, the γ- and (ω−2)-methyl branched compounds **112** and **113** were not produced under the conditions of isoleucine feeding, and therefore, their biosynthetic origin remained elusive.

#### Feeding of [^2^H_10_]leucine

Feeding of [^2^H_10_]leucine was performed to investigate the biosynthetic origin of the (ω−1)-methyl branched FAMEs. Incorporation was observed for a series of (ω−1)-methyl branched FAs including isovaleric acid, 5-methylhexanoic acid, 7-methyloctanoic acid, 9-methyldecanoic acid (Figures S3A and S3B of Supporting Information File 1), and 11-methyldodecanoic acid, all with high incorporation rates (>70%). The uptake of deuterated leucine for the last compound was observable by a shift of the molecular ion from *m*/*z* = 186 to *m*/*z* = 195. The fragment ions of the McLafferty rearrangement (*m*/*z* = 60) and the β-cleavage (*m*/*z* = 73) remained unchanged, whereas fragment ions arising from cleavage of the terminal isopropyl group (*m*/*z* = 43 and *m*/*z* =143) shifted to *m*/*z* = 50 and *m*/*z* = 145 in agreement with the deuterium labelling of this portion of the molecule.

The labelling was also introduced into the *iso*-odd FAMEs **102** and **103** (Figures S3C to S3F of Supporting Information File 1) and the higher homologue methyl 15-methylhexadecanoate (not shown). Methyl 15-methylhexadecanoate was only found during feeding of [^2^H_10_]leucine, similar to the formation of methyl 14-methylhexadecanoate found only during feeding of [^2^H_10_]isoleucine.

#### Feeding of [^2^H_8_]valine

Feeding of [^2^H_8_]valine resulted in the incorporation of the isotopic labelling into its transamination-oxidative decarboxylation product isobutyric acid (**49**) and the *iso*-even FAMEs methyl 12-methyltridecanoate (**100**) and methyl 14-methylpentadecanoate (**101**) with high incorporation rates (>50%), as indicated by the increase of the molecular ions by 7 amu in combination with the overall expected fragmentation pattern (Figure S4 of Supporting Information File 1). The transamination product of valine, 2-oxoisovaleric acid, can be used by most organisms for the biosynthesis of the leucine precursor 2-oxoisocaproic acid. The enzymes for catalysis of this pathway are encoded in the genome of *M. aurantiaca*, but the pathway seemed not to be active under the experimental conditions, judged by the fact that no incorporation of [^2^H_8_]valine into the *iso*-odd FAMEs was observed. Furthermore, no uptake of labelling from [^2^H_10_]leucine into the *iso*-even FAMEs was found, which also rules out a similar formation of the higher homologue 2-oxo-5-methylcaproic acid from 2-oxoisocaproic acid. These results also demonstrate that the FAs in *M. aurantiaca* are not subject to α-oxidation, a process in which FAs are oxidatively degraded by one carbon.

#### Feeding of [^2^H_5_]sodium propionate

To investigate the biosynthetic origin of the α- and γ-methyl branches, a feeding experiment with [^2^H_5_]sodium propionate was performed. This compound was expected to be carboxylated to yield methylmalonyl-CoA, which would serve as an elongation unit for the introduction of methyl branches. Alternative mechanisms could include the chain elongation with malonyl-CoA, followed by methylation with SAM by catalysis of a *C*-methyltransferase. Indeed the incorporation of isotopic labelling into the α- and γ-methyl branched FAMEs was observed with high incorporation rates (>90%, Figure S5 of Supporting Information File 1). Incorporation into methyl 2,11-dimethyldodecanoate (**25**) and its higher homologue **26** was observable by an increase of the molecular ion by 3 amu, while the fragment ions at *m*/*z* = 88 and *m*/*z* = 101, indicative of an α-methyl branch, shifted to *m*/*z* = 91 and *m*/*z* = 104. The incorporation of only three deuterium atoms from [^2^H_5_]sodium propionate is in agreement with the biosynthesis of FAs (Scheme 1): One deuterium is lost during carboxylation of propionyl-CoA to yield methylmalonyl-CoA, the second is possibly exchanged with water due to the C,H-acidity of malonyl-CoA derivatives, but it is lost, at the latest, in the dehydration of the 3-hydroxy-2-methylacyl-S~ACP to the respective 2-methyl-2-enoyl-S~ACP intermediate. The incorporation of [^2^H_5_]sodium propionate was also observed for the γ-methyl branched compounds represented by methyl 4,11-dimethyldodecanoate (**110**). The uptake of deuterium was in the first instance visible by an increase of the molecular ion from *m*/*z* = 242 to *m*/*z* = 245, whereas the McLafferty rearrangement and β-cleavage fragment ions were detected at *m*/*z* = 74 and *m*/*z* = 87, as for the unlabelled compound. The deuterium labelling of the γ-methyl group was indicated by a shift of the fragment ion at *m*/*z* = 115 (γ-cleavage) to *m*/*z* = 118. No incorporation was observed into the (ω−2)-methyl branches of the respective FAMEs, ruling out their alternative formation from an acetate starter in combination with an initial methylmalonyl-CoA elongation unit, instead of from isoleucine.

#### Feeding of [*methyl*-^2^H_3_]methionine

Feeding of [*methyl*-^2^H_3_]methionine was performed, first as a control experiment with respect to the biosynthetic origin of the α- and γ-methyl branches, and second, to investigate the biosynthetic origin of the methyl ester moiety of the FAMEs. The feeding experiment resulted in the incorporation into **103** (Figure S6 of Supporting Information File 1) and **97** (not shown), as indicated by a shift of the molecular ion from *m*/*z* = 256 to *m*/*z* = 259, of the McLafferty ion from *m*/*z* = 74 to *m*/*z* = 77, and of the β-cleavage fragment ion from *m*/*z* = 87 to *m*/*z* = 90. Further FAMEs were not produced during the course of this experiment. The results clearly demonstrate the formation of FAMEs from methionine via SAM as the methyl donor for the methylation of FAs.

## Conclusion

In summary, the headspace extracts from *M. aurantiaca* contain unbranched even and odd FAMEs that are made from an acetyl-CoA or propionyl-CoA starter. In particular the even FAMEs are very widespread in nature. Several α-methyl branched FAMEs were also present that were previously described from *Chitinophaga* [14]. These compounds can be synthesised by incorporation of a final methylmalonyl-CoA elongation unit. When methylmalonyl-CoA is used as the penultimate building block followed by malonyl-CoA, the synthesis results in the formation of γ-methyl branched FAs that upon *O*-methylation yield the respective FAMEs. Only one such compound has previously been found in nature represented by methyl 4-methyloctanoate, and this is a constituent of the pheromone blend of the date palm fruit stalk borer *Oryctes elegans* [22], whereas the related FAMEs **89**–**94** emitted by *M. aurantiaca* are all new natural products. The use of alternative starter units from branched amino acids for the biosynthesis of FAMEs was demonstrated in feeding experiments. Although the respective FAs are widespread in nature, only a few reports of these FAMEs exist, e.g., the isoleucine-derived compounds **95** and **96** occur in the volatile fraction from *Medicago sativa* [23], the valine-derived FAME **99** is known from *Capsicum annuum* [24], and the leucine-derived compound **8** was previously found in *Stigmatella aurantiaca* [13], whereas the compounds **102** and **103** (from leucine), and **98** (from valine) have not been described before. The same is true for all FAMEs described here that are derived from a branched amino acid and that are in addition α- or γ-methyl-branched, and there is only one report on such unusual FAs with a similar methyl branching pattern from *Bacillus* [25]. The biosynthesis of these compounds was established in feeding experiments with [^2^H_5_]sodium propionate and [*methyl*-^2^H_3_]methionine, which allowed us to distinguish between two possible pathways, i.e., the incorporation of methylmalonyl-CoA elongation units, or the alternative incorporation of malonyl-CoA elongations units followed by methylation with SAM. The experiments clearly demonstrated the usage of methylmalonyl-CoA building blocks, whereas the feeding experiment with [*methyl*-^2^H_3_]methionine gave proof for the origin of the methyl ester functions by SAM-dependent methylation of the respective FAs.

## Supporting Information

Supporting Information contains experimental details for the syntheses and analytical data of all synthesized compounds, the tabulated full results of the headspace analyses, and mass spectra of labelled FAMEs obtained during feeding experiments together with the mass spectra of the unlabelled compounds for comparison.

File 1 Experimental details and analytical data.
