# Supplementary material for: Novel fatty acid methyl esters from the actinomycete Micromonospora aurantiaca
Source: Beilstein J Org Chem. 2011 Dec 20;7:1697–712. doi: 10.3762/bjoc.7.200 (PMC3252875; doi:10.3762/bjoc.7.200)
Supplement: File 1 — Experimental details and analytical data. [file Beilstein_J_Org_Chem-07-1697-s001.pdf]

Supporting Information

for

Novel fatty acid methyl esters from the actinomycete *Micromonospora aurantiaca*

Jeroen S. Dickschat*, Hilke Bruns and Ramona Riclea

Address: Institut für Organische Chemie, Technische Universität Braunschweig, Hagenring 30, 38106 Braunschweig, Germany

Email: Jeroen S. Dickschat - j.dickschat@tu-braunschweig.de

* Corresponding author

Experimental details and analytical data

Table S1: Compounds identified in the headspace extract of *M. aurantiaca*.

Compound[a]	*I*[b]	Ident.[c]	1.[d]	2.[d]
3-Hydroxybutan-2-one (**44**)		ms, syn	xx	x
3-Methylbutan-1-ol (**47**)		ms, syn	xxx	xx
2-Methylbutan-1-ol (**48**)		ms, syn	xx	xx
Methyl 2-methylbutyrate (**3**)		ms, syn	x	x
3-Hydroxypentan-2-one (**45**)	803	ms, syn	x	x
2-Hydroxypentan-3-one (**46**)	809	ms, syn	x	x
2-Methylpropanoic acid (**49**)	813	ms	xxx	xxx
Methylpyrazine (**64**)	822	ms, syn	x	x
Butyric acid (**57**)	840	ms	x	x
2,2-Dimethylpropanoic acid (**52**)	841	ms	x	x
3-Methylbutyric acid (**50**)	870	ms	xxx	xxx
2,5-Dimethylpyrazine (**65**)	912	ms, syn	xx	xx
2-Methylbutyric acid (**51**)	922	ms	xxx	xxx
Pentanoic acid (**58**)	925	ms	x	x
3-Methylbut-2-enoic acid (**53**)	927	ms	x	x
2-Methylbut-2-enoic acid (**54**)	939	ms	x	x
Methyl furan-2-carboxylate (**75**)	976	ms, syn	x	x
Hexanoic acid (**59**)	993	ms	x	x
Trimethylpyrazine (**66**)	1003	ms, syn	x	x
2-Acetyl-5-methylfuran (**76**)	1036	ms, syn	x	x
5-Methylhexanoic acid (**55**)	1055	ms	x	x
2-Acetylpyrrole (**69**)	1060	ms, syn	x	x
Methyl 2-methylheptanoate (**13**)	1062	ms, inc	x	x
4-Methylhexanoic acid (**56**)	1064	ms	x	x
2-Ethyl-3,6-dimethylpyrazine (**67**)	1078	ms, syn	x	x
Heptanoic acid (**60**)	1081	ms	x	x
2-Ethyl-3,5-dimethylpyrazine (**68**)	1085	ms, syn	x	x
Linalool (**80**)	1098	ms, syn	x	x
2-Phenylethanol (**70**)	1112	ms, syn	xx	xx
Phenylacetone (**71**)	1127	ms, syn	x	x
Methyl phenylacetate (**73**)	1177	ms, syn	x	x
Octanoic acid (**61**)	1179	ms	x	x

Methyl salicylate (**74**)	1192	ms, syn	x	x
Decanal (**77**)	1203	ms, syn	x	x
Methyl nonanoate (**86**)	1223	ms, syn	x	x
1-Phenylbutan-2-one (**72**)	1224	ms, syn	x	x
Methyl 2-methylnonanoate (**14**)	1259	ms, inc	x	x
Nonanoic acid (**62**)	1269	ms	x	x
Methyl 8-methylnonanoate (**98**)	1286	ms, inc	x	x
Methyl decanoate (**82**)	1322	ms, syn	x	x
7-Methyloctan-4-olide (**78**)	1323	ms, syn	x	x
Methyl 4,8-dimethylnonanoate (**106**)	1336	ms, inc	x	x
Methyl 2-methyldecanoate (**10**)	1357	ms, inc, syn	x	x
Nonan-4-olide (**79**)	1361	ms, syn	x	x
Decanoic acid (**63**)	1364	ms	x	x
Methyl 4-methyldecanoate (**89**)	1375	ms, inc	x	x
Methyl 9-methyldecanoate (**8**)	1385	ms, inc, syn	x	x
Methyl 8-methyldecanoate (**95**)	1392	ms, inc, syn	x	x
6,10-Dimethylundecan-2-one	1402	ms, syn	x	x
Methyl 2,9-dimethyldecanoate (**24**)	1419	ms, inc, syn	x	x
Methyl undecanoate (**87**)	1421	ms, syn	x	x
Methyl 4,9-dimethyldecanoate (**109**)	1437	ms, inc	x	x
Methyl 4,8-dimethyldecanoate (**112**)	1441	ms, syn	x	x
6,10-Dimethylundeca-5,9-dien-2-one (**81**)	1450	ms, syn	x	x
Methyl 2-methylundecanoate (**15**)	1456	ms, inc	x	x
Methyl 4-methylundecanoate (**92**)	1473	ms, inc	x	x
Methyl 10-methylundecanoate (**99**)	1484	ms, inc	x	x
Methyl 2,10-dimethylundecanoate (**104**)	1517	ms, inc	x	x
Methyl dodecanoate (**83**)	1520	ms, syn	x	x
Methyl 4,8-dimethylundecanoate (**114**)	1525	ms, syn	x	x
Methyl 4,10-dimethylundecanoate (**107**)	1534	ms, inc	x	x
Methyl 2-methyldodecanoate (**11**)	1555	ms, inc	x	x
Methyl 4-methyldodecanoate (**90**)	1572	ms, inc	x	x
Methyl 11-methyldodecanoate (**102**)	1584	ms, inc	x	x
Methyl 10-methyldodecanoate (**96**)	1591	ms, inc	x	x
Methyl 2,11-dimethyldodecanoate (**25**)	1617	ms, inc	x	x

Methyl 4,8-dimethyldodecanoate (**115**)	1618	ms	x	x
Methyl tridecanoate (**88**)	1620	ms, syn	x	x
Methyl 4,11-dimethyldodecanoate (**110**)	1633	ms, syn	x	x
Methyl 4,10-dimethyldodecanoate (**113**)	1641	ms, inc	x	x
Methyl 2-methyltridecanoate (**16**)	1653	ms, inc	x	x
Methyl 3,7,11-trimethyldodecanoate	1660	ms, inc	x	x
Methyl 4-methyltridecanoate (**93**)	1670	ms, inc	x	x
Methyl 12-methyltridecanoate (**100**)	1683	ms, inc	x	x
Methyl 8-ethyl-4-methyldodecanoate (**116**)	1713	ms	x	x
Methyl 2,12-dimethyltridecanoate (**105**)	1716	ms, inc	x	x
Methyl tetradecanoate (**84**)	1720	ms, syn	x	x
Methyl 4,12-dimethyltridecanoate (**108**)	1733	ms, inc	x	x
Methyl 2-methyltetradecanoate (**12**)	1753	ms, inc	x	x
Methyl 4-methyltetradecanoate (**91**)	1770	ms, inc	x	x
Methyl 13-methyltetradecanoate (**103**)	1783	ms, inc	x	x
Methyl 12-methyltetradecanoate (**97**)	1790	ms, inc	x	x
Methyl 2,13-dimethyltetradecanoate (**26**)	1816	ms, inc	x	x
Methyl 4,13-dimethyltetradecanoate (**111**)	1833	ms, inc	x	x
Methyl 2-methylpentadecanoate (**17**)	1852	ms, inc	x	x
Methyl 4-methylpentadecanoate (**94**)	1869	ms, inc	x	x
Methyl 14-methylpentadecanoate (**101**)	1883	ms, inc	x	x
Methyl hexadecanoate (**85**)	1918	ms, syn	x	x

[a]Unidentified compounds, compounds originating from the medium, and artifacts are not listed. [b]Retention indices ***I*** were determined from a homologous series of alkanes (C8–C36). [c]Compound identification was based on the mass spectrum (ms), comparison of the retention index to tabulated data from the literature (ri), comparison to a synthetic reference compound (syn), or an increment system for retention indices (inc). [d]Results from two different headspace extracts. Relative amounts of the volatile components are indicated by x: 0–2%, xx: 2–8%, xxx: > 8% of total area in the gas chromatogram.

Table S2: Determination of $FG(n)_{\text{FAME, HP-5 MS}}$ from a homologous series of unbranched FAMEs.

Compound	I[a]	$N(n)$[b]	n[c]	$FG(n)_{\text{FAME, HP-5 MS}}$[d]
Methyl hexanoate	924	600	6	324
Methyl heptanoate	1023	700	7	323
Methyl octanoate	1123	800	8	323
Methyl nonanoate (**86**)	1223	900	9	323
Methyl decanoate (**82**)	1322	1000	10	322
Methyl undecanoate (**87**)	1421	1100	11	321
Methyl dodecanoate (**83**)	1520	1200	12	320
Methyl tridecanoate (**88**)	1620	1300	13	320
Methyl tetradecanoate (**84**)	1720	1400	14	320
Methyl pentadecanoate	1819	1500	15	319
Methyl hexadecanoate (**85**)	1918	1600	16	318
Methyl octadecanoate	2118	1800	18	318

[a]Measured retention index I on a HP-5 MS column. [b]Increment for the longest alkyl chain with n carbons, $N(n) = 100\ n$. [c]Number of carbons n in the longest alkyl chain. [d]Increment for the functional group of a FAME on a HP-5 MS column, $FG(n)_{\text{FAME, HP-5 MS}}$.

Table S3: Calculated retention indices $I_{calc.}(n)$ for α-methyl branched FAMEs.

Compound	I^{a}	n^{b}	$I_{calc.}(n)^{c}$	$I_{calc.}(n)^{d}$
Methyl 2-methylheptanoate (**13**)	1062	7	1058	1061
Methyl 2-methylnonanoate (**14**)	1259	9	1257	1259
Methyl 2-methyldecanoate (**10**)	1357	10	1357	1357
Methyl 2-methylundecanoate (**15**)	1456	11	1456	1456
Methyl 2-methyldodecanoate (**11**)	1555	12	1556	1555
Methyl 2-methyltridecanoate (**16**)	1653	13	1655	1654
Methyl 2-methyltetradecanoate (**12**)	1753	14	1754	1752
Methyl 2-methylpentadecanoate (**17**)	1852	15	1854	1851

[a]Measured retention index *I* on a HP-5 MS column. [b]Number of carbons *n* in the longest alkyl chain. [c]Calculated retention indices after Equation 3, $Me_{\alpha} = 35$. [d]Calculated retention indices after Equation 3 and Equation 4.

Table S4: Calculated retention indices $I_{calc.}(n)$ for γ-methyl branched FAMEs.

Compound	I^{a}	n^{b}	$I_{calc.}(n)^{c}$	$I_{calc.}(n)^{d}$
Methyl 4-methyldecanoate (**89**)	1375	10	1373	1374
Methyl 4-methylundecanoate (**92**)	1473	11	1472	1473
Methyl 4-methyldodecanoate (**90**)	1572	12	1572	1572
Methyl 4-methyltridecanoate (**93**)	1670	13	1671	1671
Methyl 4-methyltetradecanoate (**91**)	1770	14	1770	1770
Methyl 4-methylpentadecanoate (**94**)	1869	15	1870	1869

[a]Measured retention index *I* on a HP-5 MS column. [b]Number of carbons *n* in the longest alkyl chain. [c]Calculated retention indices after Equation 3, $Me_{\gamma} = 51$. [d]Calculated retention indices after Equation 3 and Equation 5.

Table S5: Calculated retention indices $I_{calc.}(n)$ for ($\omega-2$)-methyl branched FAMEs.

Compound	I^a	n^b	$I_{calc.}(n)^c$
Methyl 5-methylheptanoate (**118b**)[d]	1093	7	1093
Methyl 6-methyloctanoate (**121c**)[d]	1193	8	1193
Methyl 8-methyldecanoate (**95**)	1392	10	1392
Methyl 10-methyldodecanoate (**96**)	1591	12	1591
Methyl 12-methyltetradecanoate (**97**)	1790	14	1889

[a]Measured retention index *I* on a HP-5 MS column. [b]Number of carbons *n* in the longest alkyl chain. [c]Calculated retention indices after Equation 3, $Me_{\omega-2} = 70$. [d]Not produced by *M. aurantiaca*, intermediates in the syntheses of **95** and **112**.

Table S6: Calculated retention indices $I_{calc.}(n)$ for ($\omega-1$)-methyl branched FAMEs.

Compound	I^a	n^b	$I_{calc.}(n)^c$
Methyl 6-methylheptanoate (**118a**)[d]	1086	7	1086
Methyl 8-methylnonanoate (**98**)	1286	9	1285
Methyl 9-methyldecanoate (**8**)	1385	10	1385
Methyl 10-methylundecanoate (**99**)	1484	11	1484
Methyl 11-methyldodecanoate (**102**)	1584	12	1584
Methyl 12-methyltridecanoate (**100**)	1683	13	1683
Methyl 13-methyltetradecanoate (**103**)	1783	14	1782
Methyl 14-methylpentadecanoate (**101**)	1883	15	1882

[a]Measured retention index *I* on a HP-5 MS column. [b]Number of carbons *n* in the longest alkyl chain. [c]Calculated retention indices after Equation 3, $Me_{\omega-1} = 63$. [d]Not produced by *M. aurantiaca*, intermediate in the synthesis of **8**.

Table S7: Calculated retention indices $I_{calc.}(n)$ for α- and (ω-1)-methyl branched FAMEs.

Compound	I^a	n^b	$I_{calc.}(n)^c$
Methyl 2,9-dimethyldecanoate (**24**)	1419	10	1420
Methyl 2,10-dimethylundecanoate (**104**)	1517	11	1519
Methyl 2,11-dimethyldodecanoate (**25**)	1617	12	1618
Methyl 2,12-dimethyltridecanoate (**105**)	1716	13	1716
Methyl 2,13-dimethyltetradecanoate (**26**)	1816	14	1815

[a]Measured retention index *I* on a HP-5 MS column. [b]Number of carbons *n* in the longest alkyl chain. [c]Calculated retention indices after Equation 3 and Equation 4, $Me_{\omega-1}$ = 63.

Table S8: Calculated retention indices $I_{calc.}(n)$ for γ- and (ω-1)-methyl branched FAMEs.

Compound	I^a	n^b	$I_{calc.}(n)^c$
Methyl 4,8-dimethylnonanoate (**106**)	1336	9	1338
Methyl 4,9-dimethyldecanoate (**109**)	1437	10	1437
Methyl 4,10-dimethylundecanoate (**107**)	1534	11	1536
Methyl 4,11-dimethyldodecanoate (**110**)	1633	12	1635
Methyl 4,12-dimethyltridecanoate (**108**)	1733	13	1734
Methyl 4,13-dimethyltetradecanoate (**111**)	1833	14	1833

[a]Measured retention index *I* on a HP-5 MS column. [b]Number of carbons *n* in the longest alkyl chain. [c]Calculated retention indices after Equation 3 and Equation 5, $Me_{\omega-1}$ = 63.

Table S9: Calculated retention indices $I_{calc.}(n)$ for γ- and (ω-2)-methyl branched FAMEs.

Compound	I^a	n^b	$I_{calc.}(n)^c$
Methyl 4,8-dimethyldecanoate (**112**)	1441	10	1444
Methyl 4,10-dimethyldodecanoate (**113**)	1641	12	1642

[a]Measured retention index *I* on a HP-5 MS column. [b]Number of carbons *n* in the longest alkyl chain. [c]Calculated retention indices after Equation 3 and Equation 5, $Me_{\omega-2}$ = 70.

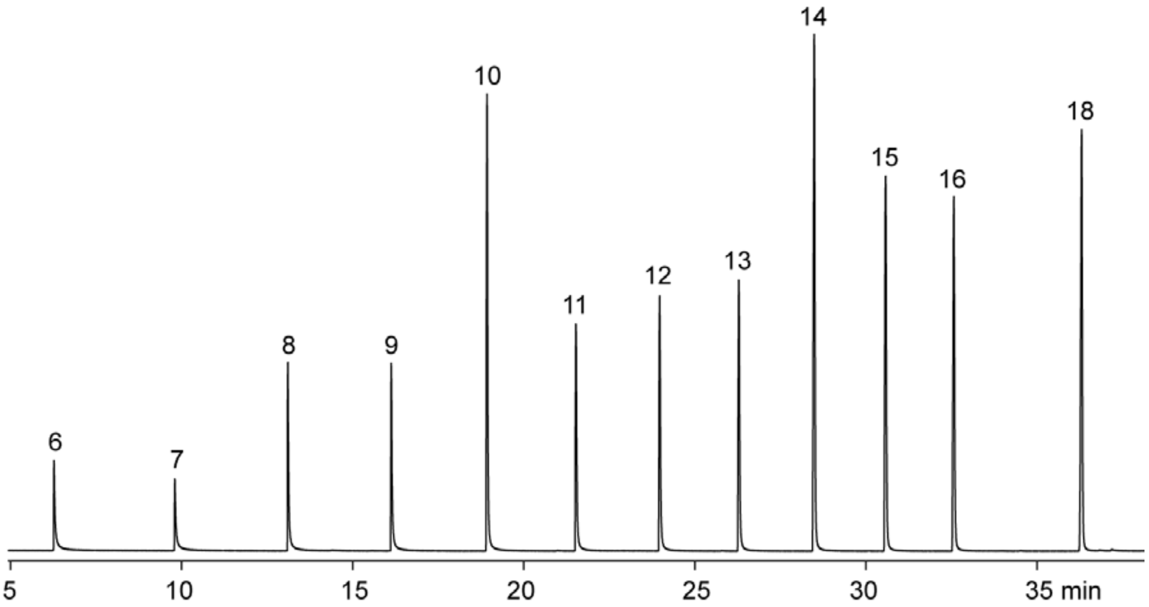

Figure S1: GC analysis of a mixture of unbranched FAMEs for the determination of the functional group increment $FG(n)_{FAME,\ HP\text{-}5\ MS}$ (Table 2 and Figure 6 of main text). Numbers above the peaks indicate the lengths of the fatty acyl chains.

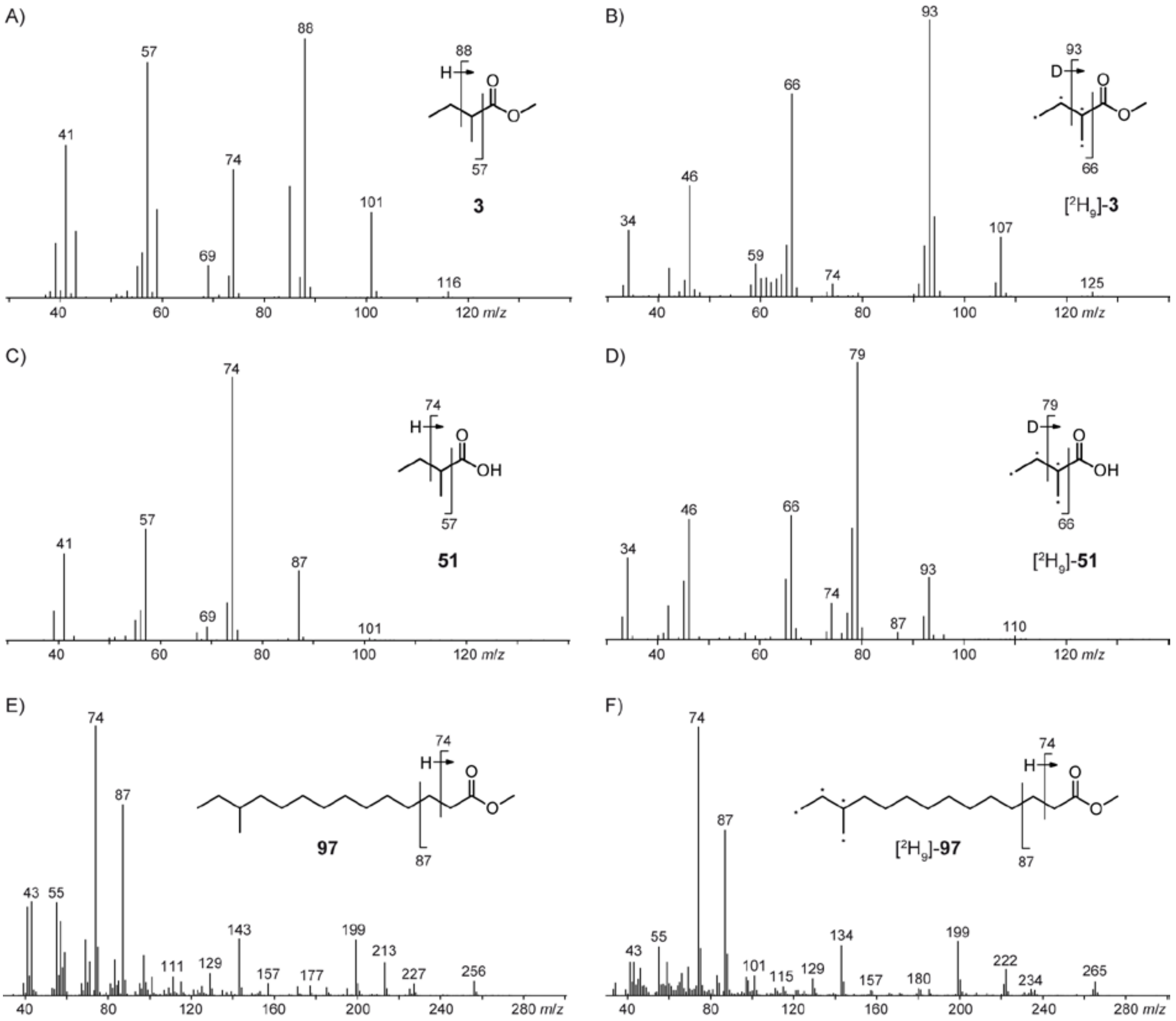

Figure S2: Mass spectra of **3** (A), of [$^2H_9$]-**3** after feeding of [$^2H_{10}$]isoleucine (B), of **51** (C), of [$^2H_9$]-**51** after feeding of [$^2H_{10}$]isoleucine, of **97** (E), and of [$^2H_9$]-**97** after feeding of [$^2H_{10}$]isoleucine. Asterisks indicate completely deuterated carbons.

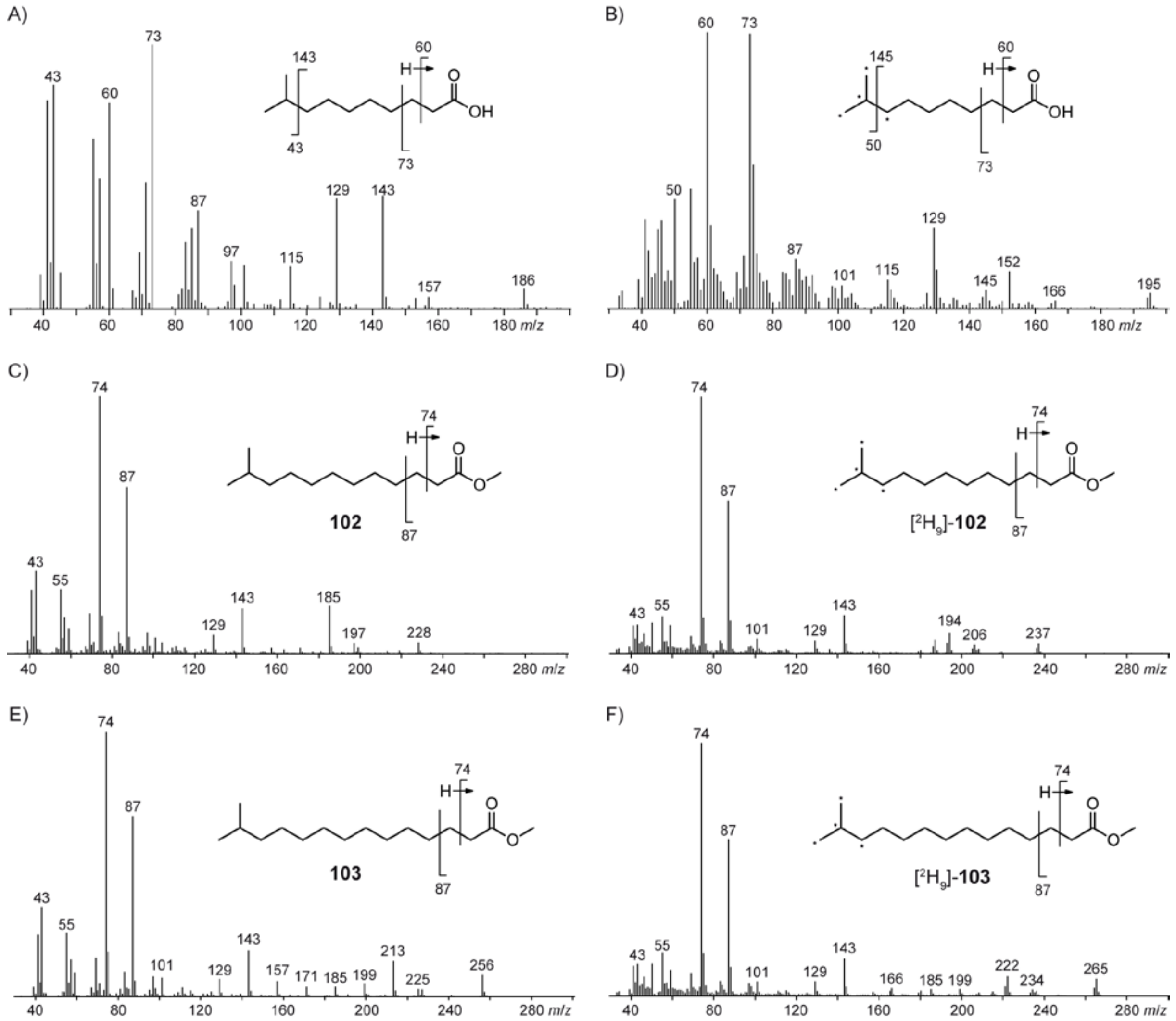

Figure S3: Mass spectra of 9-methyldecanoic acid (A), of [$^2H_9$]-9-methyldecanoic acid after feeding of [$^2H_{10}$]leucine (B), of **102** (C), of [$^2H_9$]-**102** after feeding of [$^2H_{10}$]leucine (D), of **103** (E), and of [$^2H_9$]-**103** after feeding of [$^2H_{10}$]leucine (F).

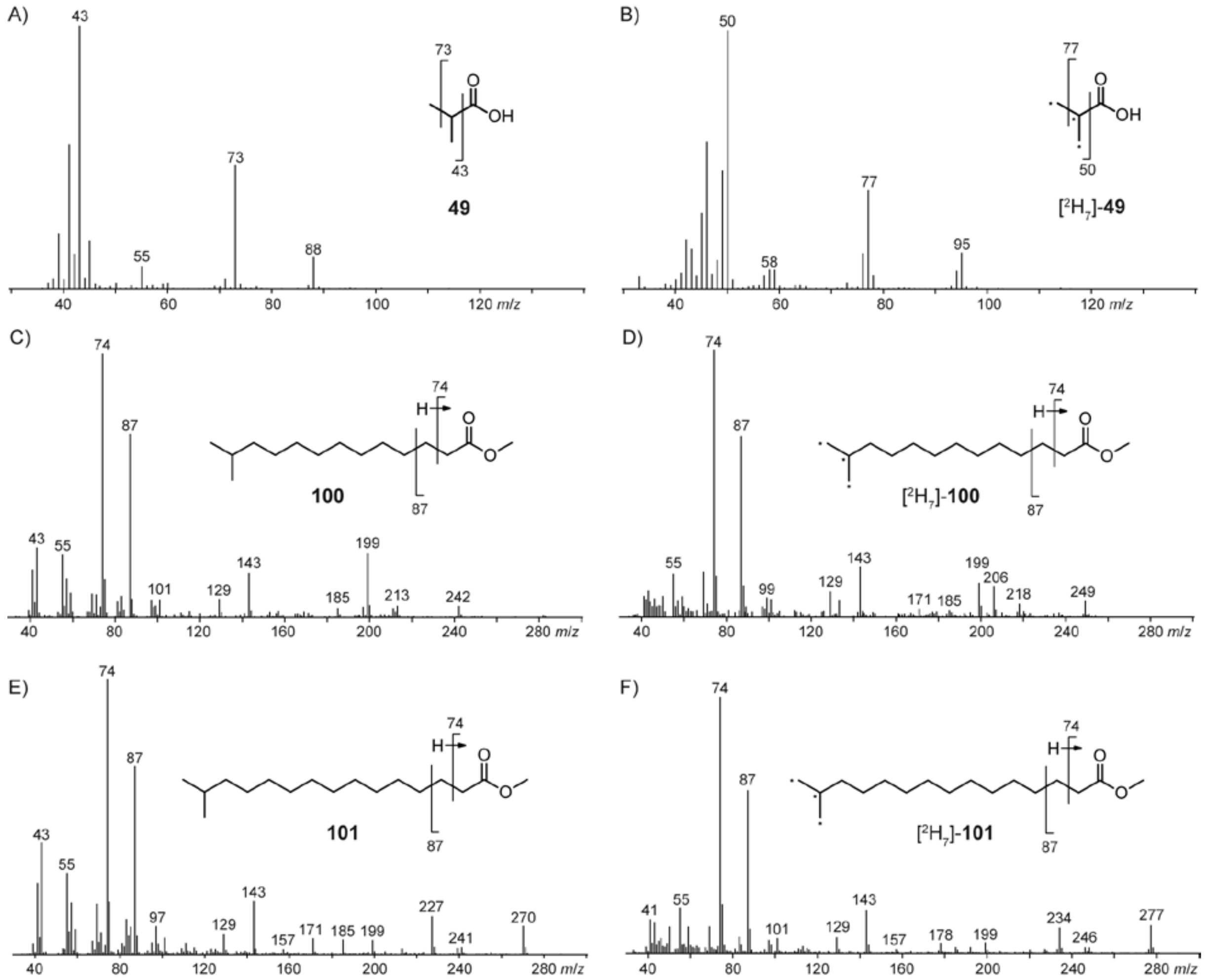

Figure S4: Mass spectra of **49** (A), of [$^{2}H_{7}$]-**49** after feeding of [$^{2}H_{8}$]valine (B), of **100** (C), of [$^{2}H_{7}$]-**100** after feeding of [$^{2}H_{8}$]valine (D), of **101** (E), and of [$^{2}H_{7}$]-**101** after feeding of [$^{2}H_{8}$]valine (F).

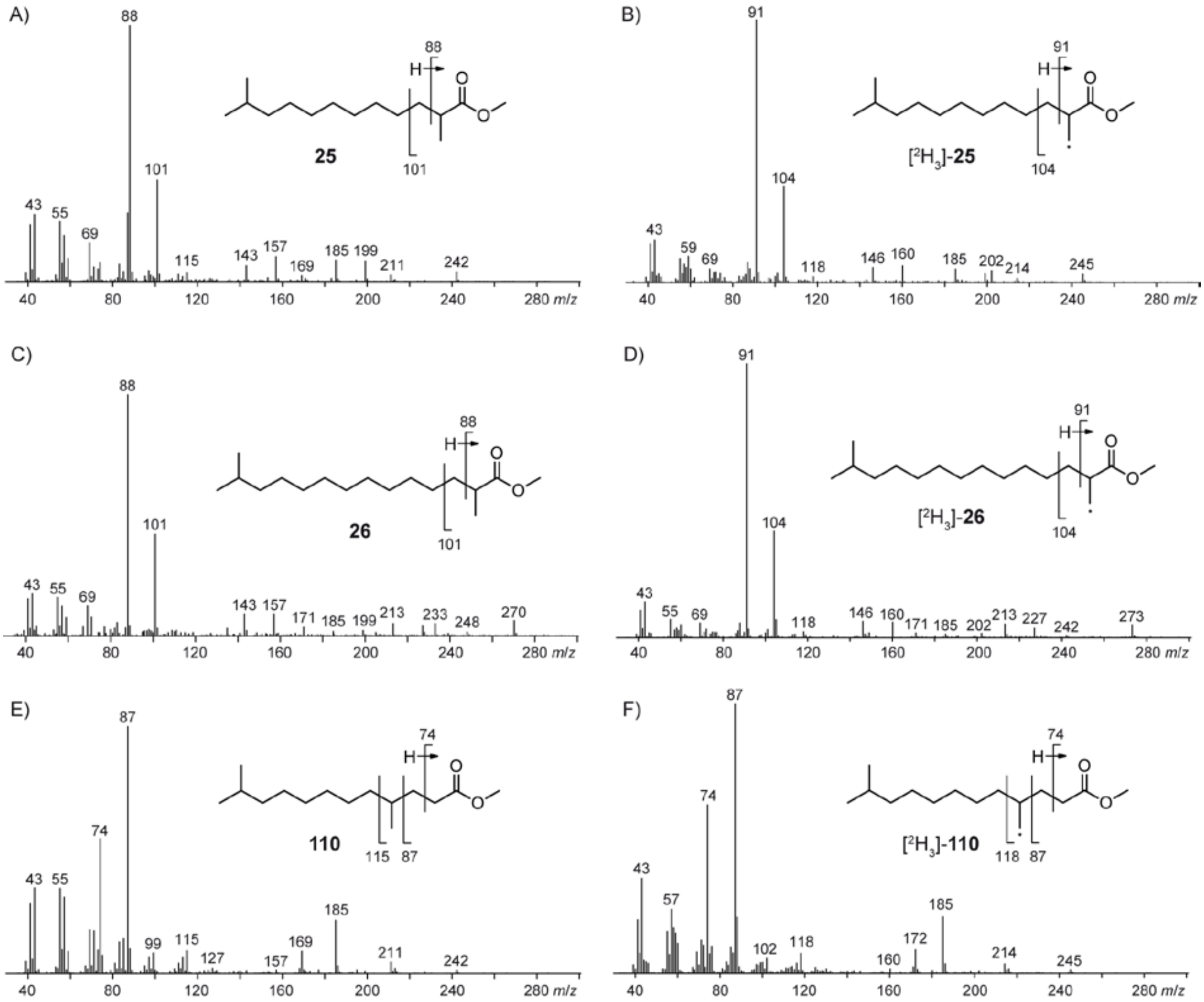

Figure S5: Mass spectra of **25** (A), of [$^{2}H_{3}$]-**25** after feeding of [$^{2}H_{5}$]sodium propionate (B), of **26** (C), of [$^{2}H_{3}$]-**26** after feeding of [$^{2}H_{5}$]sodium propionate (D), of **119** (E), and of [$^{2}H_{3}$]-**119** after feeding of [$^{2}H_{5}$]sodium propionate (F).

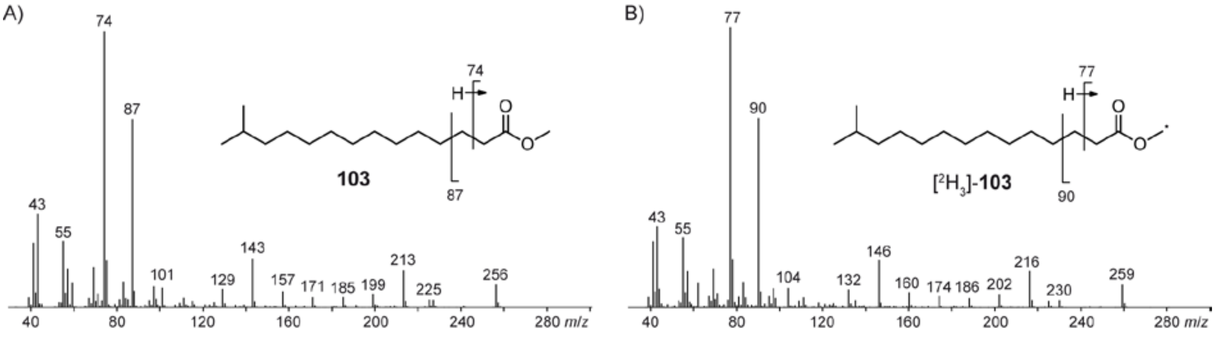

Figure S6: Mass spectra of **103** (A), and of [$^{2}H_{3}$]-**103** after feeding of [*methyl*-$^{2}H_{3}$]methionine (B).

Strains, growth conditions, and feeding experiments: *Micromonospora aurantiaca* ATCC 27029 was cultivated at 28°C in GYM 65 liquid medium (glucose: 4 g L^{-1}, yeast extract: 4 g L^{-1}, malt extract: 10 g L^{-1}, agar: 12 g L^{-1}, pH = 7.2) for 3 – 4 days. The GYM medium for the agar plates was additionally supplemented with calcium carbonate (2 g L^{-1}). The agar plates were inoculated with 1000 μL of the preculture, and spiked for feeding experiments with 2 mM of the respective deuterated precursor ([$^2H_{10}$]-L-isoleucine, [$^2H_{10}$]-D,L-leucine, [$^2H_8$]-L-valine, [*methyl*-$^2H_3$]-L-methionine, or [$^2H_5$]sodium propionate), incubated for 2 – 3 days at 37°C, and then analysed by closed-loop stripping analysis (CLSA) at 37°C.

Collection of volatiles [1]: The volatiles emitted by the agar plate cultures were collected by use of a closed-loop stripping apparatus (CLSA). Therefore, a circulating air flow was directed through a charcoal filter (Chromtech GmbH, Idstein, Precision Charcoal Filter, 5 mg) in a closed apparatus containing the agar plate, for 24 h. The charcoal filter was extracted with 20 μL of analytically pure dichloromethane and the obtained solutions were immediately analysed by GC-MS.

GC-MS: GC-MS analyses were carried out on a HP7890A GC system connected to a HP5975C mass selective detector fitted with a HP-5 fused silica capillary column (30 m, 0.22 mm i. d., 0.25 μm film, Hewlett-Packard, Wilmington, USA). Conditions were as follows: inlet pressure: 67 kPa, He 23.3 mL min^{-1}; injection volume 1 μL; injector 250°C; transfer line 300°C; electron energy 70 eV. The GC was programmed as follows: 50°C (5 min isothermic), increasing at 5°C min^{-1} to 320°C. Retention indices were determined from a homologous series of n-alkanes (C8–C32). The identification of compounds was performed by comparison of mass spectra to database spectra. Chiral GC analyses were performed by using a hydrodex-6-TBDMS fused silica capillary column (50 m, 0.25 mm i.d., 0.25 μm film, Macherey-Nagel).

General synthetic methods: Chemicals were purchased from Acros Organics (Geel, Belgium) or Sigma Aldrich Chemie GmbH (Steinheim, Germany) and used without further purification. Solvents were purified by distillation and dried according to standard methods. For all general procedures, the relative amounts of the reagents are given as equivalents (eq.) referring to the molar ratios of the compounds, and the relative amounts of the solvents are given as the final

concentrations of the transformed starting material (set to 1.0 eq.). Thin-layer chromatography was performed with 0.2 mm precoated plastic sheets Polygram® Sil G/UV254 (Machery- Nagel). Column chromatography was carried out using Merck silica gel 60 (70–200 mesh). ^{1}H NMR and ^{13}C NMR spectra were recorded on a Bruker AMX400 spectrometer and IR spectra were recorded with a Bruker Tensor 27 ATR. GC-MS analyses were carried out with an Agilent 7890A connected to an Agilent 5975C inert mass detector fitted with a HP-5 MS or BPX-5 fused silica capillary column (25 m, 0.25 mm i. d., 0.25 µm film). Instrumental parameters were (1) inlet pressure: 77.1 kPa, He 23.3 mL min^{-1}; (2) injection volume: 2 µL; (3) transfer line: 300 °C; and (4) electron energy: 70 eV. The GC was programmed as follows: 5 min at 50 °C increasing at 5 °C min^{-1} to 320 °C, and operated in splitless mode. The carrier gas was He at 1 mL min^{-1}. Retention indices *I* were determined from a homologous series of *n*-alkanes (C8–C38).

General procedure for the preparation of methyl esters via 1,4-addition to methyl acrylate [2]: To a solution of alkylmagnesium bromide, prepared from the alkyl bromide (1 M in THF, 1 eq.) and magnesium (1 eq.), DMAP (2 eq.) and $CuBr \cdot SMe_2$ (1 eq.) were added. The mixture was cooled to -78°C and a mixture of methyl acrylate (1 M in THF, 1 eq.) and TMSCl (2 M in THF, 2 eq.) was added dropwise over 30 min. After the mixture had been stirred for 3 h at -78°C, diethyl ether and HCl (2 N) were added. The aqueous phase was separated and extracted three times with diethyl ether. The combined organic layers were dried with $MgSO_4$. The pure 1,4-adduct was obtained as a colourless liquid after solvent evaporation and column chromatography.

Methyl 9-methyldecanoate (8): Yield: 5.3 g (26.5 mmol, 73%); TLC (hexane/ethyl acetate = 10:1): R_f = 0.52; GC (HP-5 MS): *I* = 1385; ^{1}H-NMR ($CDCl_3$, 400 MHz): δ = 3.66 (s, 3H, CH_3), 2.30 (t, 2H, $^{3}J_{H,H}$ = 7.6 Hz, CH_2), 1.62 (quint, 2H, $^{3}J_{H,H}$ = 7.4 Hz, CH_2), 1.51 (non, 1H, $^{3}J_{H,H}$ = 6.6 Hz, CH), 1.30-1.25 (m, 8H, 4 x CH_2), 1.17-1.12 (m, 2H, CH_2), 0.86 (d, 6H, $^{3}J_{H,H}$ = 6.6 Hz, 2 x CH_3) ppm; ^{13}C-NMR ($CDCl_3$, 100 MHz): δ = 173.9 (C=O), 51.0 (CH_3), 38.6 (CH_2), 33.7 (CH_2), 29.3 (CH_2), 28.9 (CH_2), 28.8 (CH_2), 27.6 (CH), 27.0 (CH_2), 24.6 (CH_2), 22.2 (2 x CH_3) ppm; MS (70 eV, EI): *m*/*z* (%) = 200 (3) $[M]^+$, 185 (1), 169 (6), 157 (19), 143 (16), 129 (7), 101 (13), 87 (70), 74 (100), 69 (18), 59 (20), 55 (41); IR (ATR): $1/\lambda$ = 2952 (m), 2926 (s), 2885 (m), 1742 (s),

1465 (m), 1437 (m) 1366 (m), 1248 (m), 1198 (m), 1167 (s), 1115 (w), 1012 (w), 724 (w) cm^{-1}.

Methyl 8-methyldecanoate (95): Yield: 2.91 g (14.5 mmol, 52%); TLC (hexane/ethyl acetate = 20:1): R_f = 0.22; GC (HP-5 MS): *I* = 1392; ^{1}H-NMR ($CDCl_3$, 400 MHz): δ = 3.66 (s, 3H, CH_3), 2.30 (t, 2H, $^3J_{H,H}$ = 7.6 Hz, CH_2), 1.62 (quint, 2H, $^3J_{H,H}$ = 7.5 Hz, CH_2), 1.37-1.23 (m, 9H, CH, 4 x CH_2), 1.17-1.04 (m, 2H, CH_2), 0.85 (t, 3H, $^3J_{H,H}$ = 7.2 Hz, CH_3), 0.84 (d, 3H, $^3J_{H,H}$ = 6.4 Hz, CH_3) ppm; ^{13}C-NMR ($CDCl_3$, 100 MHz): δ = 174.2 (C=O), 51.3 (CH_3), 36.5 (CH_2), 34.3 (CH), 34.1 (CH_2), 29.6 (CH_2), 29.4 (CH_2), 29.2 (CH_2), 26.8 (CH_2), 24.9 (CH_2), 19.1 (CH_3), 11.3 (CH_3) ppm; MS (70 eV, EI): *m*/*z* (%) = 200 (2) $[M]^+$, 171 (8), 143 (91), 139 (15), 115 (11), 97 (21), 87 (90), 74 (100), 69 (35), 59 (28) 57 (31), 55 (54), 43 (26), 41 (52); IR (ATR): $1/\lambda$ = 2956 (m), 2926 (m), 2856 (m), 1741 (s), 1461 (m), 1436 (m), 1376 (w), 1249 (m), 1197 (m), 1166 (m), 1113 (m), 1101 (w), 877 (w), 726 (w) cm^{-1}.

Methyl-4,8-dimethylundecanoate (114): Yield: 0.95 g (0.42 mmol, 16%); TLC (hexane/ethyl acetate = 20:1): R_f = 0.23; GC (HP-5 MS): *I* = 1525; ^{1}H-NMR ($CDCl_3$, 400 MHz): δ = 3.67 (s, 3H, CH_3), 2.38-2.24 (m, 2H, CH_2), 1.71-1.61 (m, 1H, CH), 1.49-1.36 (m, 3H, CH, CH_2), 1.33-1.19 (m, 8H, 4 x CH_2), 1.13-1.04 (m, 2H, CH_2), 0.88 (t, 3H, $^3J_{H,H}$ = 7.1 Hz, CH_3), 0.87 (d, 3H, $^3J_{H,H}$ = 6.2 Hz, CH_3) 0.84 (d, 3H, $^3J_{H,H}$ = 6.6 Hz, CH_3) ppm; ^{13}C-NMR ($CDCl_3$, 100 MHz): δ = 174.6 (C=O), 51.4 (CH_3), 39.4 (CH_2), 37.3 (CH_2), 37.0 (CH_2), 32.46 (CH), 32.42 (CH), 31.96 (CH_2), 31.88 (CH_2), 24.3 (CH_2), 20.1 (CH_2), 19.7 (CH_3) 19.3 (CH_3), 14.4 (CH_3) ppm; MS (70 eV, EI): *m*/*z* (%) = 228 (1) $[M^+]$, 213 (<1), 199 (3), 171 (13), 157 (16), 155 (14), 115 (8), 97 (9), 87 (100), 74 (39), 69 (19), 55 (31), 43 (30), 41 (20); HRMS Calcd. for $C_{14}H_{28}O_2$: 228.20893; found: 228.21124; IR (ATR): $1/\lambda$ = 2955 (m), 2926 (s), 2870 (m), 1742 (s), 1460 (m), 1436 (m), 1378 (m), 1255 (w), 1194 (m), 1168 (s), 1117 (w), 1018 (w), 992 (w), 740 (w) cm^{-1}.

Methyl 6-methylheptanoate (118a): Yield: 8.4 g (53.1 mmol, 53%); TLC (hexane/ethyl acetate = 5:1): R_f = 0.36; GC (HP-5 MS): *I* = 1086; ^{1}H-NMR ($CDCl_3$, 400 MHz): δ = 3.67 (s, 3H, CH_3), 2.31 (t, 2H, $^3J_{H,H}$ = 7.5 Hz, CH_2), 1.61 (quint, 2H, $^3J_{H,H}$ = 7.5 Hz, CH_2), 1.53 (non, 1H, $^3J_{H,H}$ = 6.6 Hz, CH), 1.35-1.27 (m, 2H, CH_2), 1.21-1.15 (m, 2H, CH_2), 0.87 (d, 6H, $^3J_{H,H}$ = 6.6 Hz, 2 x CH_3) ppm; ^{13}C-NMR ($CDCl_3$,

100 MHz): δ = 174.3 (C=O), 51.4 (CH_3), 38.5 (CH_2), 34.1 (CH_2), 27.8 (CH), 26.9 (CH_2), 25.1 (CH_2), 22.5 (2 x CH_3) ppm; MS (70 eV, EI): *m*/*z* (%) = 158 (1) $[M]^+$, 143 (3), 127 (14), 115 (18), 109 (21), 87 (85), 83 (30), 82 (23), 74 (100), 69 (16), 59 (32), 57 (18), 55 (60); IR (ATR): $1/\lambda$ = 2953 (m), 2869 (m), 1740 (s), 1464 (m), 1436 (m), 1366 (m), 1239 (m), 1197 (m), 1168 (s), 1111 (w), 996 (w), 882 (w), 741 (w) cm^{-1}.

Methyl 5-methylheptanoate (118b): Yield: 6.73 g (42.51 mmol, 66%); TLC (hexane/ethyl acetate = 20:1): R_f = 0.18; GC (HP-5 MS): *I* = 1093; ^{1}H-NMR ($CDCl_3$, 400 MHz): δ = 3.66 (s, 3H, CH_3), 2.29 (t, 2H, $^3J_{H,H}$ = 7.5 Hz, CH_2), 1.72-1.53 (m, 2H, CH_2), 1.40-1.28 (m, 3H, CH, CH_2), 1.18-1.09 (m, 2H, CH_2), 0.86 (d, 3H, $^3J_{H,H}$ = 6.5 Hz, CH_3), 0.86 (t, 3H, $^3J_{H,H}$ = 7.3 Hz, CH_3) ppm; ^{13}C-NMR ($CDCl_3$, 100 MHz): δ = 174.0 (C=O), 51.1 (CH_3), 35.9 (CH_2), 34.2 (CH_2), 34.0 (CH), 29.1 (CH_2), 22.4 (CH_2), 18.8 (CH_3), 11.1 (CH_3) ppm; MS (70 eV, EI): *m*/*z* (%) = 158 (<1) $[M]^+$, 143 (1), 129 (12), 115 (19), 109 (16), 101 (19), 87 (33), 74 (100), 69 (34), 59 (19), 55 (24), 41 (25); IR (ATR): $1/\lambda$ = 2957 (m), 2931 (m), 2874 (m), 1740 (s), 1460 (m), 1436 (m), 1377 (w), 1361 (w), 1244 (m), 1170 (s), 1110 (m), 1020 (w), 983 (w), 862 (w), 740 (w) cm^{-1}.

Methyl 6-methyloctanoate (121c): Yield: 8.0 g (46.3 mmol, 65%); TLC (hexane/ethyl acetate = 10:1): R_f = 0.38; GC (HP-5 MS): *I* = 1193; ^{1}H-NMR ($CDCl_3$, 400 MHz): δ = 3.66 (s, 3H, CH_3), 2.31 (t, 2H, $^3J_{H,H}$ = 7.5 Hz, CH_2), 1.68-1.53 (m, 2H, CH_2), 1.41-1.24 (m, 5H, 2 x CH_2, CH), 1.18-1.06 (m, 2H, CH_2), 0.85 (t, 3H, $^3J_{H,H}$ = 8.0 Hz, CH_3), 0.84 (d, 3H, $^3J_{H,H}$ = 7.3 Hz, CH_3) ppm; ^{13}C-NMR ($CDCl_3$, 100 MHz): δ = 173.9 (C=O), 51.2 (CH_3), 36.1 (CH_2), 34.1 (CH), 34.0 (CH_2), 29.3 (CH_2), 26.5 (CH_2), 25.1 (CH_2), 19.0 (CH_3), 11.2 (CH_3) ppm; MS (70 eV, EI): *m*/*z* (%) = 172 (<1) $[M]^+$, 157 (1), 143 (10), 123 (18), 115 (20), 96 (33), 87 (72), 83 (39), 74 (100), 69 (22), 59 (26), 55 (54), 43 (20), 41 (45); IR (ATR): $1/\lambda$ = 2957 (m), 2931 (m), 2872 (m), 1742 (s), 1460 (m), 1436 (m) 1376 (w), 1198 (m), 1168 (s), 1112 (m), 1011 (w), 822 (w), 734 (w) cm^{-1}.

Methyl 5-methyloctanoate (127): Yield: 6.4 g (36.9 mmol, 44%); TLC (hexane/ethyl acetate = 20:1): R_f = 0.20; GC (BPX-5): *I* = 1190; ^{1}H-NMR ($CDCl_3$, 400 MHz): δ = 3.66 (s, 3H, CH_3), 2.29 (t, 2H, $^3J_{H,H}$ = 7.7 Hz, CH_2), 1.72-1.53 (m, 2H, CH_2), 1.46-1.37 (m, 1H, CH), 1.36-1.21 (m, 2H, 2 x CH_2), 1.17-1.06 (m, 2H, CH_2), 0.88 (t, 3H, $^3J_{H,H}$ =

7.0 Hz, CH_3), 0.86 (d, 3H, $^3J_{H,H}$ = 6.6 Hz, CH_3) ppm; ^{13}C-NMR ($CDCl_3$, 100 MHz): δ = 174.1 (C=O), 51.3 (CH_3), 39.1 (CH_2), 36.4 (CH_2), 34.4 (CH_2), 32.2 (CH), 22.4 (CH_2), 20.0 (CH_2), 19.4 (CH_3), 14.2 (CH_3) ppm; MS (70 eV, EI): *m*/*z* (%) = 172 (<1) $[M]^+$, 157 (1), 141 (5), 129 (38), 123 (13), 101 (19), 97 (20), 87 (34), 74 (100), 69 (35), 59 (24), 55 (33), 43 (38), 41 (39); IR (ATR): $1/\lambda$ = 2955 (m), 2928 (m), 2872 (m), 1741 (s), 1460 (m), 1437 (m), 1378 (w), 1361 (w), 1248 (m), 1198 (m), 1169 (s), 1112 (m), 1015 (w), 874 (w), 742 (w) cm^{-1}.

General procedure for the preparation of alcohols via reduction: A solution of the ester (0.8 M in Et_2O, 1 eq.) was added to a suspension of $LiAlH_4$ (0.2 M in Et_2O, 0.75 eq.). After being heated under reflux for 12 h the mixture was cooled to 0°C and H_2O was added slowly until the H_2 formation stopped. One spatula of $MgSO_4$ was added and the mixture was stirred vigorously for 10 min. The precipitate was filtered off and the filter cake was washed excessively with Et_2O. After solvent evaporation and column chromatography on silica gel the pure alcohol was afforded as a colourless liquid.

6-Methylheptan-1-ol (119a): Yield: 5.72 g (43.9 mmol, 91%); TLC (hexane/ethyl acetate = 2:1): R_f = 0.27; GC (BPX-5): *I* = 1050; ^{1}H-NMR ($CDCl_3$, 400 MHz): δ = 3.61 (t, 2H, $^3J_{H,H}$ = 6.7 Hz, CH_2), 2.47 (s br, 1H, OH), 1.56 (quint, 2H, $^3J_{H,H}$ = 7.0 Hz, CH_2), 1.53 (non, 1H, $^3J_{H,H}$ =6.6 Hz, CH), 1.35-1.27 (m, 4H, 2 x CH_2), 1.44-1.20 (m, 2H, CH_2), 0.87 (d, 6H, $^3J_{H,H}$ = 6.7 Hz, 2 x CH_3) ppm; ^{13}C-NMR ($CDCl_3$, 100 MHz): δ = 62.7 (CH_2), 38.9 (CH_2), 32.7 (CH_2), 27.8 (CH), 27.1 (CH_2), 26.0 (CH_2), 22.5 (2 x CH_3) ppm; MS (70 eV, EI): *m*/*z* (%) = 130 (<1) $[M]^+$, 97 (26), 84 (23), 83 (8), 70 (32), 69 (84), 68 (26), 57 (41), 56 (100), 55 (91), 53 (9); IR (ATR): $1/\lambda$ = 3322 (m br), 2953 (s), 2928 (s), 2866 (m), 1465 (m), 1384 (w), 1366 (w), 1053 (s), 1029 (m), 985 (w), 726 (w) cm^{-1}.

5-Methylheptan-1-ol (119b): Yield: 4.54 g (34.20 mmol, 83%); GC (BPX-5): *I* = 1057; ^{1}H-NMR ($CDCl_3$, 400 MHz): δ = 3.61 (dt, 2H, $^3J_{H,H}$ = 6.4 Hz, 5.3 Hz, CH_2), 2.61 (t, 1H, $^3J_{H,H}$ = 5.2 Hz, OH), 1.60-1.49 (m, 2H, CH_2), 1.41-1.26 (m, 5H, CH, 2 x CH_2), 1.19-1.09 (m, 2H, CH_2), 0.86 (t, 3H, $^3J_{H,H}$ = 7.3 Hz, CH_3), 0.85 (d, 3H, $^3J_{H,H}$ = 6.3 Hz, CH_3) ppm; ^{13}C-NMR ($CDCl_3$, 100 MHz): δ = 62.7 (CH_2), 36.3 (CH_2), 34.3 (CH), 33.0 (CH_2), 29.3 (CH_2), 23.2 (CH_2), 19.0 (CH_3), 11.2 (CH_3) ppm; MS (70 eV, EI): *m*/*z* (%) =

130 (<1) $[M]^+$, 112 (<1), 97 (8), 84 (24), 83 (97), 70 (45), 69 (21), 56 (38), 55 (100), 43 (19), 41 (52), 39 (16); IR (ATR): $1/\lambda$ = 3323 (m br), 2959 (m), 2930 (s), 2871 (m), 1460 (m), 1377 (m), 1124 (w), 1054 (m), 927 (w), 769 (w), 645 (w) cm^{-1}.

5-Methyloctan-1-ol (128): Yield: 0.94 g (6.52 mmol, 91%); TLC (hexane/ethyl acetate = 5:1): R_f = 0.18; GC (BPX-5): *I* = 1078; ^{1}H-NMR ($CDCl_3$, 400 MHz): δ = 3.64 (t, 2H, $^3J_{H,H}$ = 6.6 Hz, CH_2), 1.59 (s br, 1H, OH), 1.57-1.51 (m, 2H, CH_2), 1.44-1.21 (m, 7H, CH, 3 x CH_2), 1.17-1.04 (m, 2H, CH_2), 0.88 (t, 3H, $^3J_{H,H}$ = 7.0 Hz, CH_3), 0.85 (d, 3H, $^3J_{H,H}$ = 6.5 Hz, CH_3) ppm; ^{13}C-NMR ($CDCl_3$, 100 MHz): δ = 63.0 (CH_2), 39.3 (CH_2), 36.8 (CH_2), 33.1 (CH_2), 32.4 (CH), 23.2 (CH_2), 20.1 (CH_2), 19.6 (CH_3), 14.3 (CH_3) ppm; MS (70 eV, EI): *m*/*z* (%) = 144 (<1) $[M]^+$, 126 (<1), 111 (3), 97 (22), 84 (41), 83 (98), 70 (32), 69 (33), 56 (46), 55 (100), 43 (49), 41 (43); IR (ATR): $1/\lambda$ = 3327 (w br), 2956 (m), 2929 (s), 2868 (m), 1461 (m), 1378 (m), 1125 (w), 1055 (m), 909 (w), 734 (s), 646 (w) cm^{-1}.

General procedure for the preparation of bromides: Bromine (1.33 eq.) was added dropwise to a solution of triphenylphosphane (0.7 M in dichloromethane, 1.33 eq.) at 0°C until the yellow colour persisted. The alcohol (in dichloromethane) was added in one batch. After being stirred for 2 h at 0°C, the reaction mixture was diluted with diethyl ether and washed with saturated $NaHSO_3$ solution to remove excess bromine. The aqueous phase was separated and extracted three times with diethyl ether. The combined organic layers were dried with $MgSO_4$, filtered and 2/3 of the solvents were evaporated. Pentane was added and the precipitated triphenylphosphane oxide was filtered off. Evaporation of the solvents and column chromatography provided the pure bromide as a colourless liquid.

1-Bromo-6-methylheptane (120a): Yield: 7.04 g (36.5 mmol, 85%); TLC (hexane/ethyl acetate = 10:1): R_f = 0.96; GC (BPX-5): *I* = 1116; ^{1}H-NMR ($CDCl_3$, 400 MHz): δ = 3.34 (t, 2H, $^3J_{H,H}$ = 6.9 Hz, CH_2), 1.79 (quint, 2H, $^3J_{H,H}$ = 7.0 Hz, CH_2), 1.46 (non, 1H, $^3J_{H,H}$ = 6.6 Hz, CH), 1.38-1.30 (m, 2H, CH_2),1.27-1.19 (m, 2H, CH_2), 1.13-1.08 (m, 2H, CH_2), 0.80 (d, 6H, $^3J_{H,H}$ = 6.7 Hz, 2 x CH_3) ppm; ^{13}C-NMR ($CDCl_3$, 100 MHz): δ = 39.1 (CH_2), 34.3 (CH_2), 33.2 (CH_2), 28.7 (CH_2), 28.2 (CH), 26.8 (CH_2), 22.9 (2 x CH_3) ppm; MS (70 eV, EI): *m*/*z* (%) = 193 (<1) $[M]^+$, 149 (77), 147 (79), 137 (13), 135 (14), 109 (5), 107 (5), 97 (21), 69 (56), 57 (23), 55 (57), 43 (87), 41 (100); IR

(ATR): $1/\lambda$ = 2954 (m), 2929 (s), 2866 (m), 1464 (m), 1384 (w), 1367 (w), 1260 (w), 1230 (w), 1170 (w), 1096 (w), 1019 (w), 804 (m), 728 (w), 647 (w), 564 (m) cm^{-1}.

1-Bromo-5-methylheptane (120b): Yield: 5.66 g (29.31 mmol, 91%); TLC (pentane/diethylether = 10:1): R_f = 0.86; GC (BPX-5): *I* = 1122; ^{1}H-NMR ($CDCl_3$, 400 MHz): δ = 3.41 (t, 1H, $^3J_{H,H}$ = 6.9 Hz, CH_2), 1.88-1.80 (m, 2H, CH_2), 1.52-1.38 (m, 2H, CH_2), 1.37-1.27 (m, 3H, CH, CH_2), 1.18-1.08 (m, 2H, CH_2), 0.86 (t, 3H, $^3J_{H,H}$ = 7.3 Hz, CH_3), 0.86 (d, 3H, $^3J_{H,H}$ = 6.5 Hz, CH_3) ppm; ^{13}C-NMR ($CDCl_3$, 100 MHz): δ = 36.6 (CH_2), 34.2 (CH), 33.9 (CH_2), 33.1 (CH_2), 29.4 (CH_2), 25.7 (CH_2), 19.1 (CH_3), 11.3 (CH_3) ppm; MS (70 eV, EI): *m*/*z* (%) = 192 (<1) $[M]^+$, 165 (8), 163 (8), 137 (99), 135 (100), 123 (5), 121 (5), 109 (5), 107 (6), 97 (5), 95 (4), 83 (59), 57 (52), 55 (77), 41 (68), 39 (26); IR (ATR): $1/\lambda$ = 2960 (s), 2931 (m), 2871 (m), 1460 (m), 1378 (w), 1251 (w), 1202 (w), 976 (w), 770 (w), 732 (w), 648 (m), 564 (m) cm^{-1}.

1-Bromo-3-methylpentane (120c): Yield: 13.8 g (83.3 mmol, 71%); TLC (hexane/ethyl acetate = 10:1): R_f = 0.86; GC (BPX-5): *I* = 911; ^{1}H-NMR ($CDCl_3$, 400 MHz): δ = 3.37 (ddd, 1H, $^3J_{H,H}$ = 9.9 Hz, 8.1 Hz, 5.9 Hz, C*H*H), 3.40 (dt, 1H, $^3J_{H,H}$ = 9.8 Hz, 7.6 Hz, CH*H*), 1.93-1.83 (m, 1H C*H*H), 1.67 (dtd, 1H, $^3J_{H,H}$ = 13.7 Hz, 7.8 Hz, 5.9 Hz, CH*H*), 1.56 (oct, 1H, $^3J_{H,H}$ = 6.5 Hz, CH), 1.42-1.32 (m, 2H, C*H*H), 1.39-1.26 (m, 1H, CH*H*), 0.89 (t, 3H, $^3J_{H,H}$ = 7.4 Hz, CH_3), 0.88 (d, $^3J_{H,H}$ = 6.6 Hz, CH_3) ppm; ^{13}C-NMR ($CDCl_3$, 100 MHz): δ = 39.6 (CH_2), 33.2 (CH), 32.2 (CH_2), 28.9 (CH_2), 18.4 (CH_3), 11.1 (CH_3) ppm; MS (70 eV, EI): *m*/*z* (%) = 165 (<1) $[M]^+$, 164 (5) $[M-1]^+$, 137 (4), 135 (4), 109 (6), 107 (6), 85 (99), 84 (45), 69 (42), 57 (100), 55 (85), 41 (78), 39 (33); IR (ATR): $1/\lambda$ = 2962 (m), 2927 (m), 2874 (m), 1461 (m), 1379 (w), 1255 (m), 1215 (w), 1154 (w), 1038 (w), 1002 (w), 965 (w), 877 (w), 776 (w), 643 (m), 566 (m) cm^{-1}.

1-Bromo-2-methylpentane (126): Yield: 14.7 g (89.3 mmol, 76%); TLC (hexane/ethyl acetate = 10:1): R_f = 0.92; GC (BPX-5): *I* = 903; ^{1}H-NMR ($CDCl_3$, 400 MHz): δ = 3.40 (dd, 1H, $^3J_{H,H}$ = 4.9 Hz, $^2J_{H,H}$ = 9.8 Hz, C*H*H), 3.32 (dd, 1H, $^3J_{H,H}$ = 6.2 Hz, $^2J_{H,H}$ = 9.8 Hz, CH*H*), 1.80 (oct, 1H, $^3J_{H,H}$ = 6.4 Hz, CH), 1.47-1.46 (m, 4H, 2 x CH_2), 1.01 (d, 3H, $^3J_{H,H}$ = 6.7 Hz, CH_3), 0.91 (t, 3H, $^3J_{H,H}$ = 7.1 Hz, CH_3) ppm; ^{13}C-NMR ($CDCl_3$, 100 MHz): δ = 41.5 (CH_2), 37.1 (CH_2), 34.9 (CH), 20.0 (CH_2), 18.7

(CH_3), 14.1 (CH_3) ppm; MS (70 eV, EI): *m/z* (%) = 165 (<1) $[M]^+$, 164 (2) $[M-1]^+$, 123 (6), 121 (6), 95 (4), 93 (4), 86 (7), 85 (100), 71 (18), 69 (12), 57 (9), 55 (18), 43 (62), 41 (49), 39 (25); IR (ATR): $1/\lambda$ = 2959 (s), 2929 (m), 2872 (m), 1460 (m), 1379 (m), 1322 (w), 1247 (w), 1228 (m), 947 (w), 845 (w), 813 (w), 739 (w), 650 (s), 619 (m), 553 (w) cm^{-1}.

2-Bromo-6-methylnonane (131): Yield: 1.03 g (4.58 mmol, 87%); TLC (pentane/diethyl ether = 10:1): R_f = 0.88; GC (BPX-5): *I* = 1257; ^{1}H-NMR ($CDCl_3$, 400 MHz): δ = 4.14 (sextt, 1H, $^3J_{H,H}$ = 6.6 Hz, $^4J_{H,H}$ = 1.8 Hz, CH), 1.87-1.73 (m, 3H, CH, CH_2), 1.71 (d, 3H, $^3J_{H,H}$ = 7.0 Hz, CH_3), 1.49-1.21 (m, 6H, 3 x CH_2), 1.17-1.05 (m, 2H, CH_2), 0.88 (t, 3H, $^3J_{H,H}$ = 7.0 Hz, CH_3), 0.86 (d, 3H, $^3J_{H,H}$ = 6.6 Hz, CH_3) ppm; ^{13}C-NMR ($CDCl_3$, 100 MHz): δ = 52.0 (CH), 41.5 (CH_2), 39.3 (CH_2), 36.3 (CH_2), 32.4 (CH), 26.5 (CH_3), 25.3 (CH_2), 20.1 (CH_2), 19.6 (CH_3), 14.4 (CH_3) ppm; MS (70 eV, EI): *m/z* (%) = 221 (<1) $[M]^+$, 179 (2), 177 (2), 151 (59), 149 (60), 141 (23), 99 (18), 97 (40), 85 (78), 71 (86), 69 (51), 57 (66), 55 (100), 43 (86), 41 (68); IR (ATR): $1/\lambda$ = 2956 (s), 2926 (m), 2869 (m), 1458 (m), 1378 (m), 1230 (w), 1213 (w), 1146 (w), 1098 (w), 961 (w), 740 (w), 620 (w), 543 (m) cm^{-1}.

General procedure for α-methylation of esters: To a cooled (0°C) solution of diisopropylamine (0.13 M in THF, 1.1 eq.) *n*-butyllithium (1.6 M in Hexan, 1.1 eq.) was added slowly and stirred for 1 h at 0°C. After being cooled to -78°C the ester (1 eq.) was added and the solution was stirred for 30 min. Iodomethane was added dropwise and the reaction mixture stirred for 2 h at -78°C. The mixture was allowed to warm to room temperature, the reaction was quenched with saturated NH_4Cl solution, and the layers were separated. The aqueous layer was extracted with Et_2O, the combined organic layers were dried over $MgSO_4$, filtered, and the solvents were evaporated. Column chromatography of the residue on silica gel afforded the methylated ester as a colourless liquid.

Methyl 2-methyldecanoate (10): Yield: 5.28 g (26.4 mmol, 82%); TLC (hexane/ethyl acetate = 20:1): R_f = 0.37; GC (HP-5 MS): *I* = 1357; ^{1}H-NMR ($CDCl_3$, 400 MHz): δ = 3.67 (s, 3H, CH_3), 2.43 (sext, 1H, $^3J_{H,H}$ = 6.9 Hz, CH), 1.69-1.60 (m, 1H, C*H*H), 1.44-1.36 (m, 1H,CH*H*), 1.30-1.24 (m, 12H, 6 x CH_2), 1.14 (d, 3H, $^3J_{H,H}$ = 7.0 Hz, CH_3), 0.88 (t, 3H, $^3J_{H,H}$ = 6.8 Hz, CH_3) ppm; ^{13}C-NMR ($CDCl_3$, 100 MHz): δ = 177.3 (C=O),

51.3 (CH_3), 39.4 (CH), 33.8 (CH_2), 31.8 (CH_2), 29.5 (CH_2), 29.4 (CH_2), 29.2 (CH_2), 27.2 (CH_2), 22.6 (CH_2), 17.0 (CH_3), 14.0 (CH_3) ppm; MS (70 eV, EI): *m*/*z* (%) = 200 (<1) $[M]^+$, 157 (5), 143 (6), 101 (27), 88 (100), 69 (6), 57 (15), 55 (13); IR (ATR): $1/\lambda$ = 2925 (s), 2856 (m), 1738 (s), 1462 (m), 1435 (m), 1377 (w), 1195 (s), 1164 (s), 1092 (w), 987 (w), 835 (w), 713 (w) cm^{-1}.

Methyl 2,9-dimethyldecanoate (24): Yield: 2.29 g (10.67 mmol, 79%); TLC (hexane/ethyl acetate = 10:1): R_f = 0.50; GC (HP-5 MS): *I* = 1419; ^{1}H-NMR ($CDCl_3$, 400 MHz): δ = 3.66 (d, 3H, $^4J_{H,H}$ = 0.5 Hz, CH_3), 2.44 (sext, 1H, $^3J_{H,H}$ = 7.0 Hz, CH), 1.69-1.60 (m, 1H, C*H*H), 1.51 (non, 1H, $^3J_{H,H}$ = 6.6 HZ, CH), 1.44-1.36 (m, 1H, CH*H*), 1.31-1.24 (m, 8H, 4 x CH_2), 1.17-1.12 (m, 5H, CH_2, CH_3), 0.86 (d, 6H, $^3J_{H,H}$ = 6.6 Hz, 2 x CH_3) ppm; ^{13}C-NMR ($CDCl_3$, 100 MHz): δ = 177.4 (C=O), 51.4 (CH_3), 39.4 (CH), 39.0 (CH_2), 33.8 (CH_2), 29.7 (CH_2), 29.5 (CH_2), 27.9 (CH), 27.3 (CH_2), 27.2 (CH_2), 22.6 (CH_3), 17.0 (CH_3) ppm; MS (70 eV, EI): *m*/*z* (%) = 214 (5) $[M]^+$, 199 (1), 183 (4), 171 (11), 157 (18), 143 (12), 115 (4), 101 (53), 88 (100), 69 (19), 59 (21), 57 (20), 55 (28), 43 (44), 41 (46); HRMS Calcd. for $C_{13}H_{26}O_2$: 214.19328; found: 214.19503; IR (ATR): $1/\lambda$ = 2926 (s), 2856 (m), 1739 (s), 1463 (m), 1436 (m), 1366 (m), 1249 (m), 1195 (s), 1165 (s), 1090 (w), 989 (w), 835 (w), 760 (w), 724 (w) cm^{-1}.

Methyl 2,6-dimethyloctanoate (122c): Yield: 7.00 g (37.6 mmol, 85%); TLC (hexane/ethyl acetate = 20:1): R_f = 0.36; GC (BPX-5): *I* = 1228; ^{1}H-NMR ($CDCl_3$, 400 MHz): δ = 3.66 (d, 3H, $^4J_{H,H}$ = 0.9 Hz, CH_3), 2.44 (sext, 1H, $^3J_{H,H}$ = 6.9 Hz, CH), 1.69-1.58 (m, 1H, CH), 1.43-1.21 (m, 6H, 3 x CH_2), 1.14 (d, 3H, $^3J_{H,H}$ = 7.0 Hz, CH_3), 1.13-1.05 (m, 2H, CH_2), 0.85 (t, $^3J_{H,H}$ = 6.9 Hz, CH_3), 0.84 (d, 3H, $^3J_{H,H}$ = 5.8 Hz, CH_3) ppm; ^{13}C-NMR ($CDCl_3$, 100 MHz): δ = 177.2 (C=O), 51.2 (CH_3), 39.4 (CH), 36.3 (CH_2), 34.14 (CH), 34.06 (CH_2), 29.3 (CH_2), 24.6 (CH_2), 19.0 (CH_3), 17.0 (CH_3), 11.2 (CH_3) ppm; MS (70 eV, EI): *m*/*z* (%) = 186 (2) $[M]^+$, 171 (1), 157 (16), 129 (8), 115 (4), 101 (48), 97 (20), 88 (100), 69 (17), 57 (19), 55 (27), 41 (28); IR (ATR): $1/\lambda$ = 2958 (m), 2934 (m), 2874 (m), 1738 (s), 1461 (m), 1435 (m), 1377 (m), 1256 (m), 1201 (m), 1169 (s), 1150 (s), 826 (w), 764 (w), 735 (w) cm^{-1}.

General procedure for the reduction of esters to aldehydes: A solution of the ester (0.4 M in Et_2O, 1 eq.) was cooled to -78°C and DIBAH (1 M in Hexan, 1.4 eq.) was added slowly. The reaction was monitored with TLC and upon completion of the

reaction the solution was poured into an ice-cold, stirred solution of HCl (4 N). After separation of the layers the aqueous phase was extracted three times with Et_2O. The combined organic layers were dried with $MgSO_4$, filtered, and the solvents were evaporated. Column chromatography of the residue on silica gel afforded the aldehyde as a colourless liquid.

2,9-Dimethyldecanal (123a): Yield: 0.98 g (5.33 mmol, 54%); TLC (hexane/ethyl acetate = 20:1): R_f = 0.24; GC (BPX-5): *I* = 1342; ^{1}H-NMR ($CDCl_3$, 400 MHz): δ = 9.61 (d, 1H, $^3J_{H,H}$ = 2.0 Hz, CHO), 1.73-1.64 (m, 1H, CH), 1.51 (non, 1H, $^3J_{H,H}$ = 6.6 Hz, CH), 1.47-1.39 (m, 1H, CH*H*), 1.36-1.22 (m, 9H, C*H*H, 4 x CH_2), 1.18 (d, 3H, $^3J_{H,H}$ = 7.0 Hz, CH_3), 1.17-1.12 (m, 2H, CH_2), 0.86 (d, 6H, $^3J_{H,H}$ = 6.6 Hz, 2 x CH_3) ppm; ^{13}C-NMR ($CDCl_3$, 100 MHz): δ = 205.5 (CHO), 46.3 (CH), 39.0 (CH_2), 33.5 (CH_2), 29.7 (CH_2), 29.5 (CH_2), 27.9 (CH), 27.3 (CH_2), 27.1 (CH_2), 22.6 (2 x CH_3), 16.8 (CH_3) ppm; MS (70 eV, EI): *m*/*z* (%) = 184 (1) $[M]^+$, 142 (5), 126 (8), 109 (6), 95 (12), 81 (14), 71 (25), 69 (18), 58 (100), 57 (39), 55 (32), 43 (65), 41 (68); IR (ATR): $1/\lambda$ = 2952 (m), 2926 (s), 2855 (m), 2702 (w), 1729 (s), 1463 (m), 1382 (w), 1367 (w), 1132 (m), 955 (w), 920 (w), 723 (w) cm^{-1}.

2,6-Dimethyloctanal (123c): Yield: 4.55 g (29.1 mmol, 81%); TLC (hexane/ethyl acetate = 10:1): R_f = 0.45; GC (BPX-5): *I* = 1142; ^{1}H-NMR ($CDCl_3$, 400 MHz): δ = 9.62 (d, 1H, $^3J_{H,H}$ = 2.1 Hz, CH), 2.34 (sextd, 1H, $^3J_{H,H}$ = 6.8 Hz, 2.0 Hz, CH), 1.75-1.63 (m, 1H, CH), 1.40-1.26 (m, 6H, 3 x CH_2), 1.18-1.10 (m, 2H, CH_2), 1.09 (d, 3H, $^3J_{H,H}$ = 7.0 Hz, CH_3), 0.85 (t, 3H, $^3J_{H,H}$ = 8.1 Hz, CH_3), 0.84 (d, 3H, $^3J_{H,H}$ = 7.2 Hz, CH_3) ppm; ^{13}C-NMR ($CDCl_3$, 100 MHz): δ = 205.4 (CHO), 46.4 (CH), 36.6 (CH_2), 34.2 (CH), 30.9 (CH_2), 29.4 (CH_2), 24.4 (CH_2), 19.1 (CH_3), 13.4 (CH_3), 11.3 (CH_3) ppm; MS (70 eV, EI): *m*/*z* (%) = 156 (<1) $[M]^+$, 114 (7), 109 (16), 98 (16), 81 (13), 71 (22), 69 (18), 58 (100), 57 (53), 55 (30), 43 (35), 41 (53); IR (ATR): $1/\lambda$ = 2960 (m), 2931 (m), 2873 (m), 1705 (s), 1462 (m), 1417 (w), 1378 (w9, 1291 (w), 1238 (w), 1184 (m), 942 (w), 734 (w) cm^{-1}.

2-Methyldecanal (123d): Yield: 2.98 g (17.5 mmol, 76%); TLC (hexane/ethyl acetate = 20:1): R_f = 0.33; GC (BPX-5): *I* = 1273; ^{1}H-NMR ($CDCl_3$, 400 MHz): δ = 9.61 (d, 1H, $^3J_{H,H}$ = 2.0 Hz, CH), 2.33 (sextd, 1H, $^3J_{H,H}$ = 6.8 Hz, 2.0 Hz, CH), 1.73-1.64 (m, 2H, CH_2), 1.36-1.25 (m, 12H, 6x CH_2), 1.09 (d, 3H, $^3J_{H,H}$ = 7.0 Hz, CH_3), 0.88 (t, 3H, $^3J_{H,H}$

= 6.9 Hz, CH_3) ppm; ^{13}C-NMR ($CDCl_3$, 100 MHz): δ = 205.4 (CHO), 46.3 (CH), 31.8 (CH_2), 30.5 (CH_2), 29.6 (CH_2), 29.4 (CH_2), 29.2 (CH_2), 26.9 (CH_2), 22.6 (CH_2), 14.1 (CH_3), 13.3 (CH_3) ppm; MS (70 eV, EI): *m/z* (%) = 170 (<1) $[M]^+$, 128 (5), 112 (8), 95 (5), 85 (5), 81 (6), 71 (17), 58 (100), 57 (26), 55 (17), 43 (30), 41 (30); IR (ATR): 1/λ = 2924 (s), 2855 (s), 1706 (s), 1464 (m), 1417 (w), 1378 (w), 1292 (w), 1237 (w), 1184 (m), 1111 (w), 939 (w), 722 (w), 637 (w), 543 (w) cm^{-1}.

General procedure for the preparation of α,β-unsaturated methyl esters via Horner-Wadsworth-Emmons reaction: To a cooled (0°C) solution of diisopropylamine (0.1 M in THF, 1.05 eq.) *n*-butyllithium (1.6 M in Hexan, 1.05 eq.) was added slowly and the solution stirred for 30 min at 0°C. After being cooled to -78°C trimethylphosphonoacetate (1.05 eq.) was added and the solution was stirred for 1 h. The aldehyde (0.4 M in THF, 1 eq.) was added and the reaction mixture stirred for 3 h at -78°C. The mixture was allowed to warm to room temperature, the reaction was quenched with H_2O and saturated NaCl solution, and the layers were separated. The aqueous layer was extracted with ethyl acetate, the combined organic layers were dried over $MgSO_4$, filtered, and the solvents were evaporated. Column chromatography of the residue on silica gel afforded the α,β-unsaturated methyl ester as a colourless liquid.

Methyl (*E*)- and (*Z*)-4,11-dimethyldodec-2-enoate (124a): Yield: 0.38 g (1.56 mmol, 70%), diastereomeric ratio *E* : *Z* = 87 : 13.
Methyl (*Z*)-4,11-dimethyldodec-2-enoate: TLC (hexane/ethyl acetate = 30:1): R_f = 0.22; GC (BPX-5): *I* = 1587; ^{1}H-NMR ($CDCl_3$, 400 MHz): δ = 5.97 (dd, 1H, $^3J_{H,H}$ = 11.5 Hz, 10.3 Hz, =CH), 5.71 (dd, 1H, $^3J_{H,H}$ = 11.5 Hz, $^4J_{H,H}$ = 0.9 Hz, =CH), 3.70 (s, 3H, CH_3), 3.5-3.43 (m, 1H, CH), 1.51 (non, 1H, $^3J_{H,H}$ = 6.6 Hz, CH), 1.36-1.23 (m, 10H, 5 x CH_2), 1.16-1.11 (m, 2H, CH_2), 1.00 (d, 3H, $^3J_{H,H}$ = 6.7 Hz, CH_3), 0.86 (d, 6H, $^3J_{H,H}$ = 6.6 Hz, 2 x CH_3) ppm; ^{13}C-NMR ($CDCl_3$, 100 MHz): δ = 166.8 (C=O), 156.6 (=CH), 117.7 (=CH), 50.9 (CH_3), 39.0 (CH_2), 37.0(CH_2), 32.7 (CH), 29.8 (CH_2), 29.7 (CH_2), 27.9 (CH), 27.4 (CH_2), 27.2 (CH_2), 22.6 (2 x CH_3), 20.3 (CH_3) ppm; MS (70 eV, EI): *m/z* (%) = 240 (36) $[M]^+$, 209 (12), 128 (38), 127 (100), 114 (30), 96 (34), 95 (49), 81 (40), 69 (29), 67 (33), 55 (47), 43 (69), 41 (61); HRMS Calcd. for $C_{15}H_{28}O_2$: 240.20893; found: 240.21014; IR (ATR): 1/λ = 2953 (m), 2925 (m), 2854 (m), 1726

(s), 1645 (m) 1462 (m), 1437 (m), 1368 (w), 1194 (s), 1174 (s), 1135 (m), 1007 (m), 932 (w), 822 (s), 724 (w) cm^{-1}; UV/VIS (CH_2Cl_2): λ_{max} (log ε) = 230 (3.28) nm.

Methyl (*E*)-4,11-dimethyldodec-2-enoate: TLC (hexane/ethyl acetate = 30:1): R_f = 0.10; GC (BPX-5): *I* = 1679; ^{1}H-NMR ($CDCl_3$, 400 MHz): δ = 6.87 (dd, 1H, $^3J_{H,H}$ = 15.7 Hz, 7.9 Hz, =CH), 5.78 (dd, 1H, $^3J_{H,H}$ = 15.7 Hz, $^4J_{H,H}$ = 1.2 Hz, =CH), 3.73 (s, 3H, CH_3), 2.34-2.24 (m, 1H, CH), 1.51 (non, 1H, $^3J_{H,H}$ = 6.6 Hz, CH), 1.36-1.25 (m, 10H, 5 x CH_2), 1.18-1.11 (m, 2H, CH_2), 1.04 (d, 3H, $^3J_{H,H}$ = 6.7 Hz, CH_3), 0.86 (d, 6H, $^3J_{H,H}$ = 6.6 Hz, 2 x CH_3) ppm; ^{13}C-NMR ($CDCl_3$, 100 MHz): δ = 167.4 (C=O), 155.1 (=CH), 119.1 (=CH), 51.4 (CH_3), 40.0 (CH_2), 36.6 (CH), 36.0 (CH_2), 29.8 (CH_2), 29.7 (CH_2), 27.9 (CH), 27.3 (CH_2), 27.1 (CH_2), 22.6 (2 x CH_3), 19.4 (CH_3) ppm; MS (70 eV, EI): *m*/*z* (%) = 240 (5) $[M]^+$, 209 (21), 185 (6), 166 (14), 128 (100), 127 (48), 110 (38), 96 (69), 95 (48), 87 (36), 81 (53), 69 (50), 67 (35), 55 (69), 43 (93), 41 (81); HRMS Calcd. for $C_{15}H_{28}O_2$: 240.20893; found: 240.21040; IR (ATR): 1/λ =2953 (m), 2926 (s), 2855 (m), 1726 (s), 1656 (m) 1464 (m), 1436 (m), 1367 (w), 1311 (w), 1270 (m), 1195 (m), 1172 (s), 1035 (w), 1015 (w), 984 (m), 863 (w), 824 (w), 724 (m) cm^{-1}; UV/VIS (CH_2Cl_2): λ_{max} (log ε) = 231 (3.16) nm.

Methyl (*E*)- and (*Z*)-4,8-dimethyldec-2-enoate (124c): Yield: 1.78 g (98.4 mmol, 65%); diastereomeric ratio *E* : *Z* = 67 : 33.

Methyl (*Z*)-4,8-dimethyldec-2-enoate: TLC (hexane/ethyl acetate = 30:1): R_f = 0.33; GC (BPX-5): *I* = 1389; ^{1}H-NMR ($CDCl_3$, 400 MHz): δ = 5.97 (ddd, 1H, $^3J_{H,H}$ = 11.4 Hz, 10.3 Hz, $^4J_{H,H}$ = 1.2 Hz, =CH), 5.71 (dd, 1H, $^3J_{H,H}$ = 11.6 Hz, $^4J_{H,H}$ = 0.6 Hz, =CH), 3.70 (s, 3H, CH_3), 3.58-3.45 (m, 1H, CH), 1.36-1.21 (m, 7H, 3 x CH_2, CH), 1.00 (d, 3H, $^3J_{H,H}$ = 6.7 Hz, CH_3), 0.84 (t, 3H, $^3J_{H,H}$ = 7.3 Hz, CH_3) 0.84-0.82 (m, 3H, CH_3) ppm; ^{13}C-NMR ($CDCl_3$, 100 MHz): δ = 166.7 (C=O), 156.5 (=CH), 117.8 (=CH), 50.8 (CH_3), 37.3 (CH_2), 36.3 (CH_2), 34.3 (CH), 32.7 (CH), 29.4 (CH_2), 24.7 (CH_2), 20.2 (CH_3), 19.1 (CH_3), 11.3 (CH_3) ppm; MS (70 eV, EI): *m*/*z* (%) = 212 (20), $[M]^+$, 197 (1), 181 (9), 151 (13), 128 (27), 127 (100), 114 (38), 113 (18), 96 (27), 95 (52), 81 (42), 67 (37), 57 (33), 55 (44), 41 (50); HRMS Calcd. for $C_{13}H_{24}O_2$: 212.17763; found: 212.17859; IR (ATR): 1/λ = 2958 (m), 2927 (m), 2872 (m), 1726 (s), 1645 (m), 1460 (m), 1437 (m), 1407 (m), 1376 (w), 1196 (s), 1174 (s), 1007 (m), 934 (w), 822 (s), 729 (w) cm^{-1}; UV/VIS (CH_2Cl_2): λ_{max} (log ε) = 229 (3.43) nm.

Methyl (*E*)-4,8-dimethyldec-2-enoate: TLC (hexane/ethyl acetate = 30:1): R_f = 0.17; GC (BPX-5): *I* = 1478; ^{1}H-NMR ($CDCl_3$, 400 MHz): δ = 6.87 (ddd, 1H, $^3J_{H,H}$ = 15.7 Hz, 7.9 Hz, $^4J_{H,H}$ = 1.8 Hz, =CH), 5.78 (dd, 1H, $^3J_{H,H}$ = 15.7 Hz, $^4J_{H,H}$ = 1.1 Hz, =CH), 3.73 (s, 3H, CH_3), 2.30 (sept, 1H, $^3J_{H,H}$ = 6.7 Hz, CH), 1.36-1.23 (m, 7H, 3 x CH_3, CH), 1.15-1.07 (m, 2H, CH_2), 1.04 (d, 3H, $^3J_{H,H}$ = 6.7 Hz, CH_3), 0.85 (t, 3H, $^3J_{H,H}$ = 7.2 Hz, CH_3), 0.83 (d, 3H, $^3J_{H,H}$ = 6.0 Hz, CH_3) ppm; ^{13}C-NMR ($CDCl_3$, 100 MHz): δ = 167.4 (C=O), 155.1 (=CH), 119.1 (=CH), 51.4 (CH_3), 36.7 (CH), 36.6 (CH_2), 36.3 (CH_2), 34.3 (CH), 29.5 (CH_2), 24.7 (CH_2), 19.4 (CH_3), 19.2 (CH_3), 11.4 (CH_3) ppm; MS (70 eV, EI): *m*/*z* (%) = 212 (3), $[M]^+$, 197 (1), 181 (24), 151 (25), 138 (20), 128 (100), 127 (66), 123 (40), 114 (42), 110 (45), 96 (70), 95 (57), 87 (52), 83 (52), 82 (52), 81 (74), 70 (41) 69 (60), 57 (67), 55 (88), 41 (77); HRMS Calcd. for $C_{13}H_{24}O_2$: 212.17763; found: 212.17966; IR (ATR): $1/\lambda$ = 2959 (m), 2928 (m), 2873 (m), 1725 (s), 1657 (m), 1460 (m), 1435 (m), 1378 (w), 1352 (w), 1269 (s), 1199 (m), 1173 (s), 1152 (m), 1016 (m), 984 (m), 865 (m), 724 (w) cm^{-1}; UV/VIS (CH_2Cl_2): λ_{max} (log ε) = 229 (3.38) nm.

Methyl (*E*)- and (*Z*)-4-methyldodec-2-enoate (124d): Yield: 2.26 g (9.97 mmol, 85%); diastereomeric ratio *E* : *Z* = 65 : 35.

Methyl (*Z*)-2-methyldodec-2-enoate: TLC (hexane/ethyl acetate = 30:1): R_f = 0.20; GC (BPX-5): *I* = 1528; ^{1}H-NMR ($CDCl_3$, 400 MHz): δ = 5.97 (dd, 1H, $^3J_{H,H}$ = 11.5 Hz, 10.3 Hz, =CH), 5.71 (dd, 1H, $^3J_{H,H}$ = 10.5 Hz, $^4J_{H,H}$ = 0.9 Hz, =CH), 3.70 (s, 3H, CH_3), 3.54-3.44 (m, 1H, CH), 1.36-1.25 (m, 14H, 7 x CH_2), 1.00 (d, 3H, $^3J_{H,H}$ = 6.7 Hz, CH_3), 0.87 (d, 3H, $^3J_{H,H}$ = 7.0 Hz, CH_3) ppm; ^{13}C-NMR ($CDCl_3$, 100 MHz): δ = 166.8 (C=O), 156.6 (=CH), 117.7 (=CH), 50.9 (CH_3), 37.0 (CH_2), 32.7 (CH), 31.9 (CH_2), 29.7 (CH_2), 29.5 (CH_2), 29.3 (CH_2), 27.3 (CH_2), 22.6 (CH_2), 20.2 (CH_3), 14.0 (CH_3) ppm; MS (70 eV, EI): *m*/*z* (%) = 226 (31) $[M]^+$, 195 (15), 152 (10), 128 (31), 127 (100), 114 (30), 96 (29), 95 (43), 87 (15), 81 (31), 69 (18), 67 (28), 55 (33), 43 (26), 41 (37); HRMS Calcd. for $C_{14}H_{26}O_2$: 226.19328; found: 226.19283; IR (ATR): $1/\lambda$ = 2955 (m), 2924 (s), 2854 (m), 1725 (s), 1645 (m), 1460 (m), 1437 (m), 1407 (m), 1194 (s), 1174 (s), 1007 (m), 931 (w), 822 (s), 723 (w) cm^{-1}; UV/VIS (CH_2Cl_2): λ_{max} (log ε) = 230 (3.36) nm.

Methyl (*E*)-2-methyldodec-2-enoate: TLC (hexane/ethyl acetate = 30:1): R_f = 0.10; GC (BPX-5): *I* = 1619; ^{1}H-NMR ($CDCl_3$, 400 MHz): δ = 6.87 (dd, 1H, $^3J_{H,H}$ = 15.7 Hz, 7.9 Hz, =CH), 5.77 (dd, 1H, $^3J_{H,H}$ = 15.7 Hz, $^4J_{H,H}$ = 1.2 Hz, =CH), 3.73 (s, 3H, CH_3),

2.34-2.24 (m, 1H, CH), 1.30-1.23 (m, 14H, 7 x CH_2), 1.04 (d, 3H, $^3J_{H,H}$ = 6.7 Hz, CH_3), 0.88 (d, 3H, $^3J_{H,H}$ = 6.9 Hz, CH_3) ppm; ^{13}C-NMR ($CDCl_3$, 100 MHz): δ = 167.4 (C=O), 155.1 (=CH), 119.1 (=CH), 51.4 (CH_3), 36.5 (CH), 36.0 (CH_2), 31.9 (CH_2), 29.6 (CH_2), 29.5 (CH_2), 29.3 (CH_2), 27.2 (CH_2), 22.6 (CH_2), 19.4 (CH_3), 14.1 (CH_3) ppm; MS (70 eV, EI): *m*/*z* (%) = 226 (3) $[M]^+$, 195 (26), 152 (24), 128 (100), 127 (51), 114 (28), 110 (31), 96 (69), 95 (40), 87 (37), 81 (46), 69 (40), 68 (29), 55 (58), 43 (39), 41 (50); HRMS Calcd. for $C_{14}H_{26}O_2$: 226.19328; found: 226.19424; IR (ATR): $1/\lambda$ =2956 (m), 2925 (s), 2854 (m), 1726 (s), 1657 (m) 1460 (m), 1435 (m), 1351 (w), 1269 (s), 1194 (m), 1172 (s), 1149 (m), 1035 (m), 1017 (m), 983 (m), 863 (m), 723 (m) cm^{-1}; UV/VIS (CH_2Cl_2): λ_{max} (log ε) = 230 (3.29) nm.

General procedure for hydrogenation of α,β-unsaturated methyl esters: Upon addition of Pt/C (5% Pt on charcoal, 0.1 eq.) the α,β-unsaturated methyl ester (0.1 M in EtOH, 1 eq.) was hydrogenated for 1 h at 25°C and an H_2 pressure of 40 bar. After filtration over celite the solvent was evaporated. Column chromatography of the residue on silica gel afforded the saturated ester as a colourless liquid.

Methyl 4-methyldodecanoate (90): Yield: 0.28 g (1.24 mmol, 92%); TLC (hexane/ethyl acetate = 30:1): R_f = 0.30; GC (HP-5 MS): *I* = 1572; ^{1}H-NMR ($CDCl_3$, 400 MHz): δ = 3.66 (s, 3H, CH_3), 2.38-2.24 (m, 2H, CH_2), 1.71-1.61 (m, 1H, CH), 1.48-1.37 (m, 2H, CH_2), 1.32-1.19 (m, 14H, 7 x CH_2), 0.88 (t, 3H, $^3J_{H,H}$ = 6.9 Hz, CH_3) 0.87 (d, 3H, $^3J_{H,H}$ = 6.3 Hz, CH_3) ppm; ^{13}C-NMR ($CDCl_3$, 100 MHz): δ = 174.6 (C=O), 51.4 (CH_3), 36.6 (CH_2), 32.4 (CH), 31.89 (2 x CH_2), 31.87 (CH_2), 29.9 (CH_2), 29.6 (CH_2), 29.3 (CH_2), 26.9 (CH_2), 22.7 (CH_2), 19.2 (CH_3), 14.1 (CH_3) ppm; MS (70 eV, EI): *m*/*z* (%) = 228 (2) $[M]^+$, 197 (11), 171 (35), 155 (21), 115 813), 87 (100), 85 (17), 74 (56), 71 (17), 57 (29), 55 (39), 43 (32), 41 (32); IR (ATR): $1/\lambda$ = 2955 (m), 2924 (s), 2854 (m), 1742 (s), 1461 (m), 1436 (m), 1378 (w), 1254 (m), 1192 (m), 1168 (s), 1018 (w), 991 (w), 722 (w) cm^{-1}.

Methyl 4,11-dimethyldodecanoate (110): Yield: 0.92 g (0.38 mmol, 91%); TLC (hexane/ethyl acetate = 20:1): R_f = 0.18; GC (HP-5 MS): *I* = 1633; ^{1}H-NMR ($CDCl_3$, 400 MHz): δ = 3.66 (s, 3H, CH_3), 2.38-2.24 (m, 2H, CH_2), 1.71-1.61 (m, 1H, CH), 1.51 (non, 1H, $^3J_{H,H}$ = 6.6 Hz, CH), 1.46-1.37 (m, 2H, CH_2), 1.29-1.25 (m, 10H, 5 x CH_2),

1.17-1.12 (m, 2H, CH_2), 0.87 (d, 3H, $^3J_{H,H}$ = 6.4 Hz, CH_3), 0.86 (d, 6H, $^3J_{H,H}$ = 6.6 Hz, 2 x CH_3) ppm; ^{13}C-NMR ($CDCl_3$, 100 MHz): δ = 174.5 (C=O), 51.4 (CH_3), 39.0 (CH_2), 36.6 (CH_2), 32.4 (CH), 31.89 (CH_2), 31.88 (CH_2), 29.9 (CH_2), 29.9 (CH_2), 27.9 (CH), 27.4 (CH_2), 26.9 (CH_2), 22.6 (2 x CH_3), 19.3 (CH_3) ppm; MS (70 eV, EI): *m*/*z* (%) = 242 (2) $[M]^+$, 211 (6), 185 (32), 169 (14), 115 (11), 99 (8), 87 (100), 74 (50), 69 (17), 57 (23), 55 (30), 43 (32), 41 (24); IR (ATR): 1/λ = 2953 (m), 2925 (s), 2854 (m), 1742 (s), 1464 (m), 1436 (m), 1381 (w), 1253 (m), 1192 (m), 1168 (s), 1019 (w), 991 (w) cm^{-1}.

Methyl 4,8-dimethyldecanoate (112): Yield: 0.28 g (1.30 mmol, 92%); TLC (hexane/ethyl acetate = 20:1): R_f = 0.31; GC (HP-5 MS): *I* = 1442; ^{1}H-NMR ($CDCl_3$, 400 MHz): δ = 3.67 (s, 3H, CH_3), 2.38-2.24 (m, 2H, CH_2), 1.71-1.62 (m, 1H, CH), 1.49-1.38 (m, 2H, CH_2), 1.37-1.21 (m, 6H, 3 x CH_2), 1.17-1.04 (m, 3H, CH_2, CH), 0.87 (d, 3H, $^3J_{H,H}$ = 6.3 Hz, CH_3), 0.85 (t, 3H, $^3J_{H,H}$ = 7.4 Hz, CH_3) 0.84 (d, 3H, $^3J_{H,H}$ = 6.1 Hz, CH_3) ppm; ^{13}C-NMR ($CDCl_3$, 100 MHz): δ = 174.5 (C=O), 51.4 (CH_3), 37.0 (CH_2), 36.8 (CH_2), 34.3 (CH), 32.4 (CH), 31.9 (CH_2), 31.8 (CH_2), 29.5 (CH_2), 24.3 (CH_2), 19.2 (CH_3), 19.1 (CH_3), 11.3 (CH_3) ppm; MS (70 eV, EI): *m*/*z* (%) = 214 (1) $[M]^+$, 185 (5), 157 (32), 141 (18), 115 (11), 97 (8), 87 (100), 74 (44), 69 (24), 57 (31), 55 (41), 43 (21), 41 (32); IR (ATR): 1/λ = 2957 (m), 2926 (m), 2872 (m), 1741 (s), 1460 (m), 1436 (m), 1378 (m), 1255 (m), 1194 (m), 1169 (s), 1116 (m), 1016 (w), 992 (w), 773 (w) cm^{-1}.

Preparation of 5-methyloctanal (129): To a cooled solution (-60°C) of oxalyl chloride (0.91 mL, 10.6 mmol) in dichloromethane (70 mL), DMSO (1.51 mL, 21.3 mmol) in dichloromethane (15 mL) was added and the solution was stirred for 10 min. The alcohol **128** (1.28 g, 8.86 mmol) in dichloromethane (15 mL) was added, and the solution stirred for 30 min. Upon the addition of NEt_3 (6.30 mL, 44.3 mmol) and stirring for another 10 min the solution was allowed to warm to room temperature, and H_2O (50 mL) was added. After separation of the layers the aqueous layer was extracted with Et_2O (3 x 100 mL) and the combined organic layers were dried with $MgSO_4$, filtered, and the solvents were evaporated. The pure compound **129** (1.09 g, 7.63 mmol, 86%) was afforded as a colourless liquid after column chromatography on silica gel.

TLC (hexane/ethyl acetate = 10:1): R_f = 0.41; GC (BPX-5): *I* = 1077; ^{1}H-NMR ($CDCl_3$, 400 MHz): δ = 9.76 (t, 1H, $^3J_{H,H}$ = 1.9 Hz, CH), 2.43-2.31 (m, 2H, CH_2), 1.73-1.54 (m, 2H, CH_2), 1.48-1.38 (m, 1H, CH), 1.37-1.22 (m, 4H, 2 x CH_2), 1.20-1.05 (m, 2H, CH_2), 0.88 (t, 3H, $^3J_{H,H}$ = 7.5 Hz, CH_3), 0.87 (d, 3H, $^3J_{H,H}$ = 6.6 Hz, CH_3) ppm; ^{13}C-NMR ($CDCl_3$, 100 MHz): δ = 202.9 (CHO), 44.1 (CH_2), 39.1 (CH_2), 36.4 (CH_2), 32.2 (CH), 20.0 (CH_2), 19.6 (CH_2), 19.4 (CH_3) 14.3 (CH_3) ppm; MS (70 eV, EI): *m*/*z* (%) = 142 (<1) $[M]^+$, 124 (15), 109 (21), 96 (20), 95 (80), 81 (60), 70 (65), 69 (63), 57 (50), 55 (100), 43 (97), 41 (80); IR (ATR): 1/λ = 2956 (s), 2927 (m), 2871 (m), 1709 (s), 1461 (m), 1412 (w), 1378 (w), 1283 (w), 1155 (w), 1117 (m), 1066 (w), 940 (m), 741 (w) cm^{-1}.

Preparation of 6-methylnonan-2-ol (130): The aldehyde **129** (1.21 g, 8.49 mmol) was added to a cooled (0°C) solution of methylmagnesium bromide (3.39 mL, 10.2 mmol) in Et_2O (20 mL). The solution was allowed to warm to room temperature and after the solution was stirred for 12 h, HCl (2 N, 40 mL) was added. Upon separation of the layers the aqueous layer was extracted with ethyl acetate (3 x 40 mL) and the solvents were evaporated. Column chromatography on silica gel afforded the alcohol **130** (0.99 g, 6.24 mmol, 74%) as a colourless liquid.
TLC (hexane/ethyl acetate = 5:1): R_f = 0.25; GC (BPX-5): *I* = 1172; ^{1}H-NMR ($CDCl_3$, 400 MHz): δ = 3.79 (sext, 1H, $^3J_{H,H}$ = 6.2 Hz, CH), 1.83 (s br, 1H, OH), 1.45-1.23 (m, 9H, CH, 4 x CH_2), 1.19 (d, 3H, $^3J_{H,H}$ = 6.2 Hz, CH_3), 1.15-1.05 (m, 2H, CH_2), 0.88 (t, 3H, $^3J_{H,H}$ = 7.0 Hz, CH_3), 0.85 (d, 3H, $^3J_{H,H}$ = 6.5 Hz, CH_3) ppm; ^{13}C-NMR ($CDCl_3$, 100 MHz): δ = 68.1 (CH), 39.7 (CH_2), 39.3 (CH_2), 37.0 (CH_2), 32.4 (CH), 23.4 (CH_2), 23.2 (CH_2), 20.1 (CH_2), 19.5 (CH_3), 14.3 (CH_3) ppm; MS (70 eV, EI): *m*/*z* (%) = 158 (<1) $[M^+]$, 143 (5), 140 (1), 112 (6), 98 (16), 97 (46), 84 (34), 70 (42), 69 (45), 55 (57), 45 (100), 43 (51), 41 (37); IR (ATR): 1/λ = 3341 (w br), 2958 (s), 2928 (s), 2869 (m), 1461 (m) 1376 (m), 1143 (w), 1116 (m), 1079 (w), 1012 (w), 936 (w), 912 (w), 740 (w) cm^{-1}.

References

1. Dickschat, J. S.; Brock, N. L.; Citron, C. A.; Tudzynski, B. *ChemBioChem* **2011**, *12,* 2088–2095. doi:10.1002/cbic.201100268

2. Horiguchi, Y.; Matsuzawa, S.; Nakamura, E.; Kuwajima, I. *Tetrahedron Lett.* **1986,** *27,* 4025–4028. doi:10.1016/S0040-4039(00)84901-1
